# Immunoreactive proteins of *Capsicum*-based spices as a threat to human health: mass spectrometry analysis and in silico mapping

**DOI:** 10.1038/s41598-023-44775-3

**Published:** 2023-10-18

**Authors:** Barbara Wróblewska, Anna Ogrodowczyk, Ewa Wasilewska

**Affiliations:** Department of Food Immunology and Microbiology, Institute of Animal Reproduction and Food Research of the Polish Academy of Sciences, 10-748 Olsztyn, Poland

**Keywords:** Immunology, Health care

## Abstract

Dietary patterns are changing severely, especially the consumption of highly processed foods with lots of spices is increasing, carrying an increased risk of immediate hypersensitivity (type I), in sensitised individuals, due to the possible presence of allergens, especially the hidden ones. Paprika is a fruit of the *Capsicum* genus, which belongs to the *Solanaceae* family and is commonly consumed fresh or as a spice. Despite recorded cases of anaphylaxis, its allergenicity has yet to be clearly investigated. In this study, we research to identify proteins that could trigger a severe allergic reaction in patients with an equivocal clinical picture. Two types of protein extracts extracted from 3 different paprika spices were immunoblotted with sera from patients with severe allergic symptoms, presumably to paprika. Proteins from the IgE reactive bands obtained were subjected to LC–MS/MS identification and then in silico analysis to assess their possible sensitising capacity and proinflammatory potential using online tools. The spices were shown to contain a number of incompletely investigated highly immunoreactive allergenic proteins, including proteins of foreign origin (contaminants), the presence of which can stimulate inflammatory mechanisms and cross-reactivity with other food allergens, which can threaten life and health and should be investigated in detail.

## Introduction

Paprika (bell pepper, *Capsicum*) is a popular plant in various parts of the world. It is cultivated for direct consumption or as a raw material that undergoes technological processing to obtain powdered spices, oleoresin extract or pure capsaicin, which are used in the food, pharmaceutical or cosmetic industries. Its anti-inflammatory, anti-aging, anti-depressive, anti-cancer, and antioxidant properties have been described^1^. However, more research is needed into its allergenicity, which is still poorly understood.

Food allergens represent a large group of still understudied compounds, often with uncharacterised biological activity, which are additionally subject to change, either naturally or during technological processing^2–4^. Although the knowledge of them is increasing and the most important ones are already listed on food labels (14 in EU countries and 8 in the USA), there are still many under-described allergenic proteins whose accidental ingestion can lead to an adverse immune reaction, especially the hidden ones whose presence is not expected^2^. The safety of food, both raw materials and products, must be ensured in the global marketplace, involving plant breeding, processing, and goods distribution. So far, the potential microbial hazards of food are well understood, but harmful contaminants can come from trace pests and pesticides, water quality, soil and post-harvest processes, as well as unintentional contaminants that may occur during the production process and may represent, for example, hidden allergens that are difficult to detect. Unintentional cross-contaminants, additives that are not declared on the product label (repackaged products or sold by weight) or whose health risks are unknown, or proteins and protein-based complexes formed during the technological processing are the most difficult to identify and quickly define. They usually pose little threat to the general public but can have serious consequences for sensitive groups of consumers, such as allergy sufferers, whose numbers are growing dramatically.

In the gut, depending on protein solubility and susceptibility to proteolysis, the ingested allergen is internalised and processed in the epithelium, and allergen-derived peptides are presented to lymphocytes, which differentiate into Th2 cells in the presence of activators (such as cytokines, TSLP and DAMP), allowing the activation, differentiation and isotype switching of allergen-specific B cells into IgE-producing plasma cells^5,6^. Allergen-specific IgE antibodies bind to mast cells and basophils, sensitising them to the allergen. On subsequent contact with the allergen, mast cells and eosinophils degranulate, causing allergic inflammation and associated symptoms. Less obvious and recognised pathways of sensitisation are also suggested^7^. The main medical marker used to determine allergy status is the level of IgE, but clinical observation does not always correlate with serological test results. A sudden reaction of the body to an allergen can lead to life-threatening anaphylactic shock, which is not always associated with high levels of specific IgE^7–9^. Such cases are sometimes referred to as idiopathic^10^. Overlapping body reactions and allergic cross-reactions cause additional difficulties in sensitised patient follow-up. Cross-reactions between allergens with highly conserved regions of amino acid sequences and similar three-dimensional structures, as in the case of pan-allergens, are becoming increasingly common and pose a threat to allergists that needs to be thoroughly investigated^11^. Characterisation of the allergenicity and immunoreactivity of food proteins and peptides provides insight into the level of risk and helps to understand the complex mechanisms that cause food allergy.

Paprika allergy is occasionally diagnosed, so paprika proteins are not among the major food allergens; however accidental ingestion of paprika by an allergic person can cause a severe body reaction, including anaphylactic shock. The scientific literature of the last decade describes cases of anaphylaxis and cross-reactivity of paprika proteins with birch, *Prunus* representants, profilins and allergenic dyes^12–15^. New paprika allergens are also being discovered and verified^16^. Three allergens, Cap a1, Cap a 2 and Cap a 7 have been already registered in the WHO/IUIS Allergen Nomenclature Database^16–18^. However, the issue remains topical and requires in-depth work, if only because of the widespread use of this raw material. Peppers in raw or processed form can be found in commonly consumed pizzas, goulash soups, meat preparations, vegetable salads, juices, or in other foods where labelling of its presence is not always mandatory. The exception is dried whole chilli peppers, the safety and specification of which is regulated by the United Nations Economic Commission for Europe (UNECE)^19^. For consumers, spices pose a particular allergy risk because they are difficult to detect in food and are highly processed. Thermal treatments, including dry steam, are commonly used in the decontamination of *Capsicum* spices, although a combination of non-thermal methods such as infrared, UV and ozonation is also used^20^. All of these treatments affect the allergenicity of proteins, and as a result of biotic and abiotic environmental stress, defence proteins are produced in the tissues, which may be the hidden allergens^11^. Apart from clinical case reports, there is very little scientific data on the immunoreactivity of paprika, which may worry given its popularity, especially as a spice. The aim of the study was to detect immunoreactive allergenic proteins of paprika spices, including possible contaminants of environmental origin that may pose an immunological threat to the body. In the spirit of limiting research on living organisms, the extensive in vitro/in silico studies were conducted to identify the potentially most immunoreactive constituents of paprika spices. Such knowledge could be invaluable for further research into paprika allergens and for the development of protective therapies for allergic individuals.

## Results

### Characteristics of protein isolates

**Figure 1:**
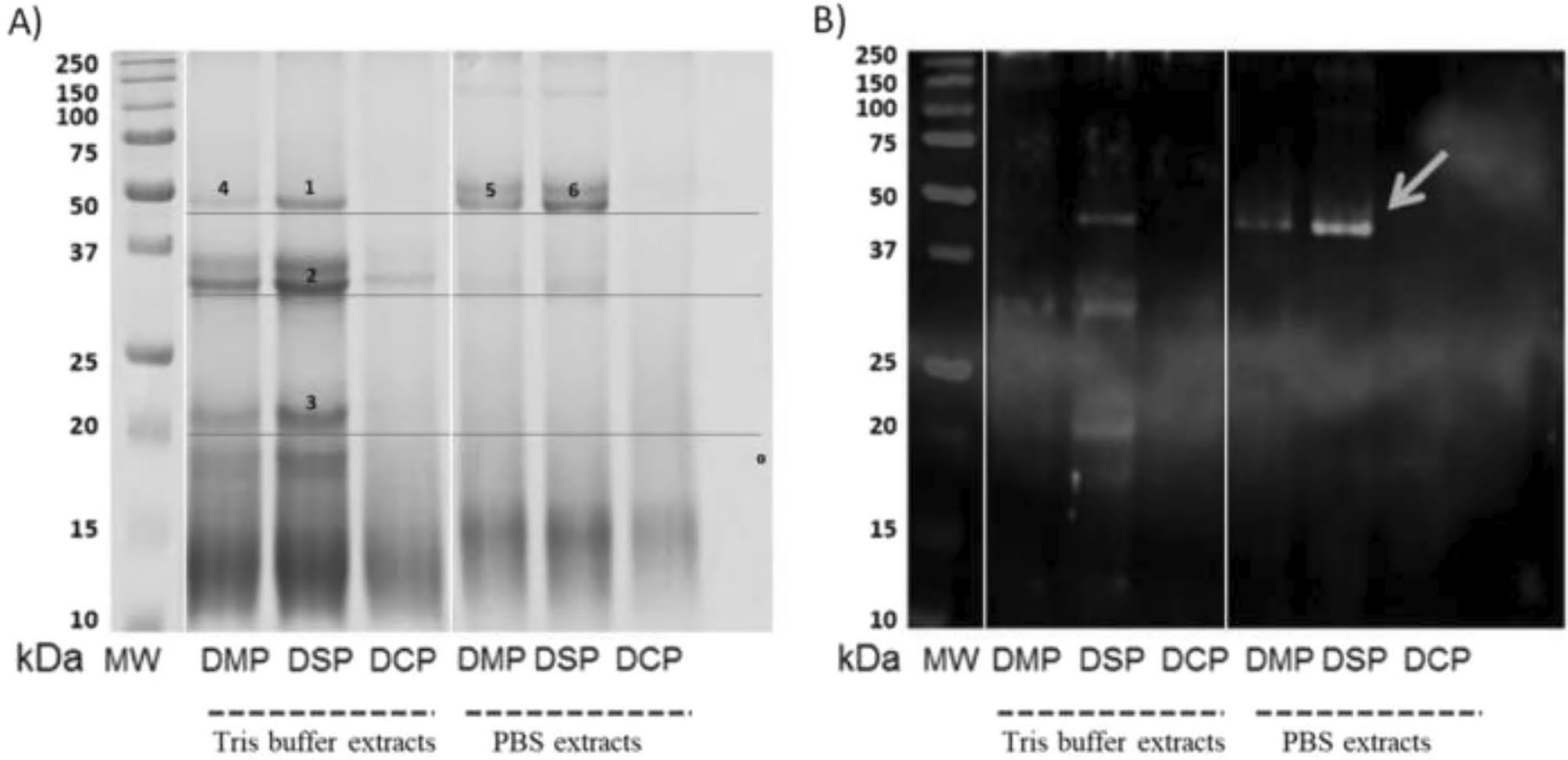
Electrophoretic separation of pepper protein extracts and their IgE-reactivity with human sera (pooled): (**A**) Tricine-SDS-PAGE electropherogram of protein isolates (20 µg); (**B**) IgE-immunoreactive spices protein immunoblot. The underline bands in figure (**A**) corresponding to IgE-immunoreactive bands in figure (**B**) were subjected to LC–MS/MS identification. *DMP* dried mild paprika, *DSP* dried spicy paprika, *DCP* dried chili paprika, *MW* molecular weight marker. Full-length gel and blot are presented in Supplementary Fig. S1.

**Figure 2:**
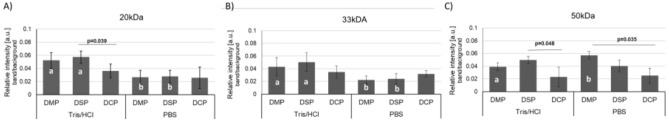
Relative signal strength for proteins of different molecular weight: (**A**) 20 kDa; (**B**) 33 kDa and (**C**) 50 kDa (ranges characterized as IgE reactive). Significant differences between the products were characterized by the Duncan test. The differences between the bands for different isolation methods were characterized by the student’s *t* test. Values *p* < 0.05 were considered significant and were marked with different letters.

Proteins were isolated from three commercial *Capsicum-*based spices (mild, chilli and spicy) using two different solvents (based on PBS and TRIS–HCl buffers) and separated by SDS-PAGE (Fig. 1A). Extracts from dried mild and spicy peppers (DMP and DSP, respectively) gave a more diverse profile than that of dried chilli pepper (DCP). For DMP and DSP, in an overall comparison of protein molecular weight profiles, the extraction of 20 kDa molecules in Tris buffer was significantly higher compared to PBS buffer (*p* < 0.05, Fig. 2A). A similar situation was observed for proteins around 33 kDa, and slightly the opposite for proteins around 50 kDa, where PBS extraction was more efficient for DMP (*p* < 0.05, Fig. 2B and C, respectively). The Tris-based method yielded the most diverse protein profile from DSP and a well-defined profile of IgE-immunoreactive epitopes compared to the PBS extract (Fig. 1B). However, the 50 kDa PBS/DSP band (extracted from DSP by PBS) gave the strongest reaction with fluorescence-labelled anti human-IgE antibodies (Fig. 1B, arrow). All IgE immunoreactive proteins were detected in the 17–50 kDa molecular mass range.

### Protein identification by LC–MS/MS analysis

The IgE immunoreactive protein bands obtained (Fig. 1) were subjected to LC–MS/MS detection. The raw data were searched by MACOT software against the Green Plant (Viridiplantae) database for all entries, to not exclude possible contaminants, and the results were then compared by BLAST with *Capsicum* taxid. The identified proteins are shown in Tables 1 and 2 (the raw mass spectrometry data generated and analysed are presented as *Supplementary data* in the supplementary material). Approximately 85% of the total identified proteins (45 out of 53) were directly assigned to the *Solanaceae* family by the MASCOT software (Table 1). Of these, approximately 40% (18 out of 45) were identified as being derived from *Capsicum annum* or *C. chinense*, and 44% from *Solanum lycopersicum*, *S. tuberosum*, or *S. peruvianum *(20 out of 44), but with high homology (78–98% identity/92–100% query cover) to *Capsicum* taxIDs. The others were assigned to *Nicotiana tomentosiformis*, *N. tabacum*, or *N. sylvestris* and their identity with *Capsicum* ranged 68–98% (85–100% query cover). In addition to proteins belonging the *Solanaceae* family, MASCOT indicated the presence of proteins belonging to other taxa that could be contaminants of the raw material (Table 2). These were proteins from *Theobroma cacao, Populus nigra, Triticum aestivum, Arabidopsis thaliana, Malus domestica, Hevea brasiliensis,* and *Dimocarpus longan*. As most of them showed high homology to *Capsicum* taxa (78–93% identity/71–100% query cover) we did not consider them as contaminants. The exception was rubber elongation factor protein from *Hevea brasiliensis* which showed low homology (43% identity) to *Capsicum* and was therefore considered a contaminant of the spices tested.

**Table 1:** *Solanaceae* proteins identified by LC–MS/MS analysis and MASCOT software in the IgE-reactive bands.

Band no.	Accession^a^	Protein name [organism, protein status]	Score^b^	Matched peptides^c^	emPAI^d^	Coverage^e^ [%]	Identity (query cover) with *Capsicum* taxa^f^ [%]—protein name//accession	MW^g^ [kDa]
2	XP_009603582	11S globulin seed storage protein 1-like [*Nicotiana tomentosiformis*]	278	5	0.25	5	74 (90)—prunin 1 Pru du 6.0101 [*Capsicum annuum*]/ XP_016570626	56.3
2, 3; 1, 4, 5, 6; 1, 3, 5; 3; 2, 4, 5	XP_009783514; XP_009624040; XP_004247523; XP_006351695; XP_006351693	11S globulin seed storage protein 2-like [*Nicotiana sylvestris*, PREDICTED; *N. tomentosiformis*; *Solanum lycopersicum*; *Solanum tuberosum*, PREDICTED]	102–510; 179–254; 122–376; 287; 104–274	3–10; 3–4; 3–8; 6; 2–5	0.25–0.7; 0.16–0.26; 0.26–0.47; 0.36; 0.17–0.37	4–12; 7–8; 8–12; 10; 7–10	81 (91)/ 80 (92)/ 80 (100)/ 81 (100)/ 78 (99)—11S globulin seed storage protein 2 [*Capsicum baccatum*]/ PHT52858	53.7
2; 1, 3, 5	XP_009624043; XP_009624041	11S globulin seed storage protein Jug r 4-like [*N. tomentosiformis*]	377; 94–235	7; 3–8	0.48; 0.17–0.27	14; 5–6	85 (96)—11S globulin seed storage protein Jug r 4-like [*C. annuum*]/ XP_016565474	52.9
1–6; 1, 3, 4, 5, 6	XP_004247735; XP_009603583	11S globulin subunit beta [*S. tuberosum*]; 11S globulin-like [*N. tomentosiformis*]	109–468; 136–426	2–7; 3–8	0.16–0.34; 0.24–0.42	5–8; 7–12	78 (96)/ 77 (96)—11S globulin seed storage protein Ana o 2.0101 [*C. annuum*]/ XP_016565958	57.9
1–6	XP_004246943	12S seed storage protein CRA1-like [*S. lycopersicum*]	130–643	4–32	0.27–0.74	9–15	87 (96)—hypothetical protein BC332_07738 [*Capsicum chinense*]/ PHU22631	52.9
3	O82013	17.3 kDa class II heat shock protein [*Solanum peruvianum*]	142	2	0.62	25	89 (100)—17.3 kDa class II heat shock protein [*C. annuum*]/ XP_016563710	17.7
3	XP_006360819	17.4 kDa class I heat shock protein [*S. tuberosum*, PREDICTED]	78	4	1.59	23	88 (100)—18.5 kDa class I heat shock protein [*C. baccatum*]/ PHT28807	18.1
3	XP_004236141	22.7 kDa class IV heat shock protein [*S. lycopersicum*]	100	2	0.48	13	84 (100)—22.0 kDa class IV heat shock protein [*C. chinense*]/ PHT97509	22.1
3	NP_001315984	2-Cys peroxiredoxin 1 [*S. lycopersicum*]	73	3	0.53	13	90 (92)—2-Cys peroxiredoxin BAS1, chloroplastic [*C. annuum*]/ XP_016543590	29.0
1	P93373	Actin-54 [*Nicotiana tabacum*]	166	5	0.75	19	98 (100)—actin-7 [*C. annuum*]/XP_016565383	41.8
1	CAI48071	Anionic peroxidase [*C. chinense*]	160	4	0.7	18	100—anionic peroxidase [*C. chinense*]/CAI48071	31.0
2	CAA63710	Annexin [*C. annuum*]	169	4	0.6	14	100—annexin [*C. annuum*]/CAA63710	35.9
1, 2	XP_004230031	Aspartyl protease AED3 [*S. lycopersicum*]	86–95	2	0.19	5	85 (100)—aspartyl protease AED3 [*C. annuum*]/XP_016557272	47.7
2	AAS20585	Basic beta-1,3-glucanase, partial [*C. annuum*]	222	3	0.61	22	99 (96) – lichenase [*C. annum*]/XP_016563240 76 (96)—basic beta-1,3-glucanase [*C. annuum*]/AAF34761	40.6 39.2
2	AAR90844	Chitinase class I, partial [*C. annuum*]	76	1	0.37	14	100—basic 30 kDa endochitinase precursor [*C. annuum*]/ NP_001311510	34.6
1, 2, 4, 5, 6	XP_009630003	Cocosin 1-like [*N. tomentosiformis*]	186–292	4–6	0.25–0.45	9–10	79 (95)—prunin 1 Pru du 6.0101 [*Capsicum annuum*]/ XP_016570626	56.3
2	XP_004230208	Desiccation-related protein PCC13-62-like [*S. lycopersicum*]	125–249	2–3	0.27	8–9	83 (98)—desiccation-related protein PCC13-62 [*C. annuum*]/ KAF3614133	37.9
1	NP_001234080	Enolase [*S. lycopersicum*]	117	2	0.19	6	98 (100)—enolase [*C. annuum*]/ XP_016542903	47.8
2, 3	CAA50750	Fibrillin [*C. annuum*]	67–332	1–9	0.13–1.59	4–39	100—fibrillin [*C. annuum*]/ CAA50750	35.3
2	XP_006362723	GDSL esterase/lipase At1g71250 [*S. tuberosum*, PREDICTED]	157	3	0.38	14	90 (99)—GDSL esterase/lipase At1g71250 [*C. annuum*]/ XP_016563440	40.8
3	AAN39918	Glutathione S-transferase [*C. annuum*]	46	1	0.18	7	100—glutathione S-transferase [*C. annuum*]/ AAN39918	24.9
3	AAD50436	Hypersensitive response assisting protein [*C. annuum*]	61–88	1–2	0.15–0.32	5–9	100—hypersensitive response assisting protein [*C. annuum*]/ AAD50436	29.9
2	XP_004247663	Late embryogenesis abundant protein 31-like [*S. lycopersicum*]	127	2	0.37	13	87 (96)—late embryogenesis abundant protein D-34 [*C. chinense*]/ PHU21880	26.7
2, 3; 2	XP_009601547; ABI73975	Late embryogenesis abundant protein D-29-like [*N. tomentosiformis*]; LEA protein 4 [*C. annuum*]	156–175; 103	3–4; 2	0.57–0.58; 1.72	13–14; 38	72 (85)/ 100—late embryogenesis abundant protein D-29 [*C. annuum*] XP_016551859	27.6
3; 1, 3, 5; 2, 3, 4, 5, 6	XP_009765013; XP_004234041; XP_006356113	Legumin B-like [*N. sylvestris*, PREDICTED; *S. lycopersicum*; *S. tuberosum*, PREDICTED]	124; 143–274; 132–233	4; 4–6; 5–7	0.35; 0.16–0.35; 0.36–0.47	9; 6–8; 6–8	77 (95)/ 82 (100)/ 82 (97)—prunin 1 Pru du 6.0101 [*C. annuum*]/ XP_016570626	56.3
3	AAT71313	Lipocalin protein [*C. annuum*]	54	1	0.22	5	100—lipocalin protein [*C. annuum*]/ AAT71313	21.3
3	ADJ57588	Mitochondrial small heat shock protein [*C. annuum*]	122	2	0.42	11	100—small heat shock protein, chloroplastic [*C. annuum*]/ NP_001311883	24.1
2	XP_004230182	NADPH-dependent aldehyde reductase 1, chloroplastic [*S. lycopersicum*]	155	3	0.4	11	89 (100)—NADPH-dependent aldehyde reductase 1, chloroplastic [Capsicum annuum]/ XP_047250615	37.8
3	XP_004251808	Oleosin 5-like [*S. lycopersicum*]	124	3	0.99	15	91 (97)—oleosin 21.2 kDa [*C. annuum*]/ PHT73078	25.4
3	AAF63519; CAI51309	Pathogenesis-related protein 10 [*C. annum*; *C. chinense*]	70; 85	1; 2	0.27; 0.37	8; 9	100—pathogenesis-related protein 10 [C. annum]/AAF63519; 100—pathogenesis-related protein 10 [C. chinense]/ CAI51309	17.3
2	CAB57457	Pectin methylesterase, partial [*N. tabacum*]	130	3	0.53	14	94 (100)—pectinesterase 2 [*C. annuum*]/ KAF3647503	29.3
2, 3	ACB30360	PGIP [*C. annuum*]	53–202	1–5	0.15–0.75	4–20	100—PGIP [*C. annuum*]/ ACB30360	29.8
1	AAC26785	Phosphoglycerate kinase precursor [*S. tuberosum*]	118	2	0.18	6	93 (100)—phosphoglycerate kinase, chloroplastic [*C. annuum*]/ PHT77520	57.1
3	CAA54961	Putative chromoplastic oxydo-reductase [*C. annuum*]	71	2	0.17	5	100—putative chromoplastic oxydo-reductase [*C. annuum*]/ CAA54961	56.8
3	CAI48023	Putative pathogenesis related protein [*C. chinense*]	105	3	1.06	22	100—putative pathogenesis related protein [*C. chinense*]/ CAI48023	17.2
1, 2	ABQ65860; XP_004250970	Serine carboxypeptidase III [*N. tabacum*]; serine carboxypeptidase-like [*S. lycopersicum, S. tuberosum,* PREDICTED]	86–161; 129	2–3; 2	0.16–0.25; 0.16	4–6; 7	83 (98)/ 85 (97)—serine carboxypeptidase 3 [*C. chinense*]/ PHU03819	57.1
5	XP_006354928	Serpin-ZX-like [*S. tuberosum*, PREDICTED]	163	4	0.48	12	91 (100)—serpin-ZX [*C. annuum*]/ XP_016568712	42.9
3	AAP57477	Small heat shock protein [*C. annuum*]	54	1	0.27	5	100—small heat shock protein [*C. annuum*]/ AAP57477	25.9
3	ABY26941	Small heat shock protein class I, partial [*C. annuum*]	77	2	1.4	26	99 (100)—17.8 kDa class I heat shock protein [*C. annuum*]/ XP_016577734	17.8
5	NP_001295320	Suberization-associated anionic peroxidase 2 precursor [*S. lycopersicum*]	232	6	0.72	19	84 (100)—suberization-associated anionic peroxidase 2 [*C. annuum*]/ PHT90648	38.5
3	AAK97184	Thaumatin-like protein [*C. annuum*]	85	1	0.17	7	100—osmotin-like protein OSML13 precursor [*C. annuum*]/ NP_001311827	26.8
3; 3	AAL35363; AFU48610	Thioredoxin peroxidase [*C. annuum; N. tabacum*]	95–127	3	0.52–1.04	12–13	100—thioredoxin peroxidase [*C. annuum*]/ AAL35363; 90 (94)—2-Cys peroxiredoxin BAS1, chloroplastic [*C. annuum*]/ XP_016543590	17.4; 29.0
1	AAB54016	Transaldolase [*S. tuberosum*]	211	4	0.42	12	89 (100)—transaldolase [*C. chinense*]/ PHT54042	48.2
2, 3	XP_009605859	Vicilin Car i 2.0101-like [*N. tomentosiformis*]	73–114	2–4	0.18–0.28	4–7	68 (97)—vicilin Jug r 2.0101 [*C. annuum*]/ XP_016567997	47.2
2; 2, 3	XP_009619507; XP_006349017	Xyloglucan endotransglucosylase/hydrolase protein 31-like [*N. tomentosiformis*; *S. tuberosum*, PREDICTED]	87; 104–123	3; 2–3	0.45; 0.28–0.45	16; 9–13	91 (93)/ 95 (95)—xyloglucan endotransglucosylase/hydrolase protein 31 [*C. annuum*]/ XP_016547781	34.2

^a^NCBI database accession, ^b^The protein score derived from the ions scores of MS report based on the calculated probability, when the significance threshold was chosen to be 0.05, score cut-off-43, ^c^Number of significant peptide matches, ^d^The Exponentially Modified Protein Abundance Index (emPAI), ^e^Coverage expressed in % number of amino acids in a specific protein sequence that were found in significant peptide matches, ^f^Percent identity and query cover of the tested protein to the best scored sequence of *Capsicum* taxa proteins deposed in NCBI database (https://www.ncbi.nlm.nih.gov), tested with The Basic Local Alignment Search Tool (BLAST). ^g^Molecular weight (MW) calculated from the UniProt database (http://www.uniprot.org/).

**Table 2:** Proteins identified by LC–MS/MS analysis and MASCOT software in the IgE-reactive bands as putative raw material contaminants.

Band no.	Accession^a^	Protein name [organism]	Score^b^	Matched peptides^c^	mPAI^d^	Coverage^e^ [%]	Identity (query cover) with *Capsicum* taxa^f^ [%]—protein name//Accession	MW^g^ [kDa]	Allergenicity hazard of proteins^h^
3	XP_007016441	17.4 kDa class I heat shock protein [*Theobroma cacao*, PREDICTED]	190	6	2.19	32	81 (100)—18.5 kDa class I heat shock protein [*C. baccatum*]/ PHT28807	18.1	*AllergenOnline*: 6 and 5/1and 0 scores, XP_007016441—62% identity (85% similar) with putative unassigned allergen (ABF21077), E = 0.22; PHT28807—29% identity (58% similar) with allergen Gly m 5, E = 0.28 *Allergome:* 1 and 1/1 score, 83 and 75% identity with putative allergen Cas s 9.0101, E < 1e-07
1	BAA33801	cytosolic phosphoglycerate kinase 1 [*Populus nigra*]	156	2	0.22	8	91 (100)—phosphoglycerate kinase, cytosolic [*C. annuum*]/ XP_016581536	42.4	*AllergenOnline*: 1 and 2/0 scores, 25 and 23% identity (55 and 54% similar) with allergen Cor a 1.0401, E = 0.61/0.98
3	P02277	histone H2A.2.2 [*Triticum aestivum*]	99	2	0.68	13	85 (82)—histone H2A.1 [Capsicum annuum]/ XP_016538626	16.1	*AllergenOnline*: 6 and 0/0 scores, 32% identity (58% similar) with putative allergen Pen c 3, E = 0.11 *Allergome:* 1 and 1/0 scores, 69 and 61% identity with putative allergen Lol p FT and Rat n trensferin, E = 16 and 58, respectively
3	CAA69025	histone H2B like protein [*Arabidopsis thaliana*]	161	4	1.21	23	93 (71)—Histone H2B.6 [*C. baccatum*]/ PHT44714	16.1	*AllergenOnline*: 8 and 23/0 scores, CAA69025—31% identity (55% similar) with allergen Lol p 5.0102, E = 0.013 and PHT44714—33% identity (62% similar) with allergen Hev b 5.0101, E = 0.13
3	XP_008343823	LOW QUALITY PROTEIN: 18.2 kDa class I heat shock protein [*Malus domestica*]	165	5	1.59	48	78 (100)—chloroplast small heat shock protein class I [*C. frutescens*]/ AAQ19680	18.2	*AllergenOnline*: 4 and 5/0 scores, XP_008343823—27% identity (59% similar) with allergen Gly m 5, E = 0.44; and AAQ19680—36% identity (60% similar) with putative unassigned allergen (ABW86979.1), E = 0.42; *Allergome:* 1 and 1/1, 83 and 72% identity with putative allergen Cas s 9.0101, E < 1e-07
3	NP_194858	N-terminal nucleophile aminohydrolases (Ntn hydrolases) superfamily protein [*A. thaliana*]	103	2	0.4	13	86 (98) proteasome subunit beta type-6 [*C. annum*]/ XP_016573160	25.1	*AllergenOnline:* 0 and 15/0 scores, XP_016573160 – 32% identity (55% similar) with allergen Zea m 12.0104, E = 0.096
2, 3	P15252	rubber elongation factor protein [*Hevea brasiliensis*]	116–138	3	1.32–1.33	32	43 (78)—stress-related protein [*C. baccatum*]/ PHT38668	27.7	*AllergenOnline:* 2/2 and 0 scores, 100 and 44% identity (100 and 72% similar) with allergens Hev b 1.0101; and 52 and 46% identity (79 and 74% similar) with allergens Hev b 3; E < 1e-7
1	ACK56136	transaldolase [*Dimocarpus longan*]	190	4	0.42	11	81 (100)—transaldolase [*Capsicum annuum*]/ XP_016554348	48.3	*AllergenOnline:* 1 and 3/0 scores, 36 and 33% identity (65 and 63% similar) with putative allergen Pen ch 35.0101, E = 0.2/0.035

^a^NCBI database accession number, ^b^The protein score derived from the ions scores of MS/MS report based on the calculated probability, when the significance threshold was chosen to be 0.05, score cut-off-43, ^c^Number of significant peptide matches, ^d^The Exponentially Modified Protein Abundance Index (emPAI), ^e^Coverage expressed in % number of amino acids in a specific protein sequence that were found in significant peptide matches, ^f^Percent identity and query cover of the tested protein to the best scored sequence of *Capsicum* taxa proteins deposed in NCBI database (https://www.ncbi.nlm.nih.gov), tested with The Basic Local Alignment Search Tool (BLAST), ^g^Molecular Weight (MW) calculated from the database used in this study (http://www.uniprot.org/), ^h^Allergenicity hazard of both proteins estimated from Allergen Online database (http://www.allergenonline.org) using the Full FASTA 36 search algorithm (E-value Cutoff = 1) and Allergome database (http://www.allergome.org) using the NCBI blastp algorithm (% identity Cutoff = 60). Only positive results are showed, for *AllergenOnline*: total scores/number of scores with > 50% identity and E < 1e-7, allergenic sequence with the best score; for *Allergome*: total scores with E-value Cutoff = 1/including scores with 70% identity, the allergenic sequence with the best score. The first results are for protein from MASCOT, the second from BLAST.

### Allergenicity assessment using online databases

Proteins identified by LC–MS/MS and BLAST software were subjected to in silico analysis to check their allergenic potential using the online allergen databases (AllergenOnline, Allergome and Allermatch). The results for the proteins indicated by MASCOT as belonging to *Solanaceae* and their *Capsicum* homologues indicated by BLAST were similar, so we have presented the results obtained for *Capsicum*. For 22 of the 45 proteins analysed, the FASTA 36 or BLASTP alignment tools used showed greater than 50% identity (79–100% similar) with allergens or putative allergens described in AllergenOnline and/or Allergome databases (Table 3). Of these 22 proteins, 16 showed greater than 70% identity with allergenic sequences, with E < 1e-7. Four of these allergens have been described as allergen or putative allergen of *Capsicum*, i.e. Cap a Glucanase, (basic beta-1,3-glucanase), Cap a 1.0101 (osmotin-like protein, allergen), the in silico generated Cap ch 17kD (submitted name: major allergen Pru ar 1) and Cap a 4 (pathogenesis-related protein 10), and two others, i.e. Sola l 4.0201 (submitted name: PR10 protein) and Sola l Peroxidase (anionic peroxidase) as putative allergens of *Solanum lycopersicum* (tomato; the *Solanaceae* family). Our proteins showed 84–100% and 70–84% sequence identity with *Capsicum* and *Solanum* allergens, respectively. In addition, the allergen prediction tools used showed alignments of *Capsicum-*derived proteins with taxonomically unrelated allergens (Sin a 2, Pers a 1.0101, Cas s 9.0101, Hev b 9 and others) that even exceeded 70% identity at E < 1e-7, indicating a high potential for allergenic cross-reactions (Table 3). A significant number of proteins (23) showed relatively low homology with known allergens and are therefore presented in the supplementary data (Supplementary Table 1S).

**Table 3:** Proteins with high allergenicity hazard—results of in silico analysis^a^.

Protein^b^	Allergen online scores^c^	Allergome scores^d^
11S globulin seed storage protein 2 [C. baccatum]/ PHT52858	66/1; 62% identity (83% similar) with allergen Ses i 6.0101, E < 1e-07	1 score; 64% identity with allergen Ses i 6.0101, E < 1e-7
11S globulin seed storage protein Ana o 2.010 [*C. annuum*]/XP_016565958	69/6 scores; 58% identity (81% similar) with putative allergen Ses i 7.0101, E < 1e-07	1 score; 60% identity with putative allergen Fag e 1, E < 1e-7
11S globulin seed storage protein Jug r 4-like [*C. annuum*]/ XP_016565474	77/14; 53% identity (83% similar) with putative allergen Ber e 2.0101, E < 1e-07	1 score; 71% identity with allergen Sin a 2, E = 015
17.8 kDa class I heat shock protein [*C. annuum*]/ XP_016577734	6/0; 20% identity (71% similar) with putative allergen Tre ke 1, E = 0.037	1 score; 70% identity with putative allergen Cas s 9.0101, E < 1e-07
18.5 kDa class I heat shock protein [*C. baccatum*]/ PHT28807	5/0; 29% identity (58% similar) with allergen Gly m 5.0101, E = 0.28	1 score; 75% identity with putative allergen Cas s 9.0101, E < 1e-07
Actin-7-like [*C. annuum*]/ XP_016565383	No matches	11/2 scores; 87% identity with in silico generated allergen Sal s alpha Actin, E = 0.0/ Asp f gamma Actin
Anionic peroxidase [*C. chinense*]/ CAI48071	No matches	2/1; 82% identity with putative allergen Sola l Peroxidase, E < 1e-7
Basic 30 kDa endochitinase precursor [*C. annuum*]/ NP_001311510	14/7; 75% identity (90% similar with putative allergen Pers a 1.0101, E < 1e-07	20/12; 76% identity with putative allergen Pers a 1.0101, E < 1e-7/Tri a Endochitinase, Mus a 2, Hev b 11.0102, Mus xp 2
Basic beta-1,3-glucanase [*C. annuum*]/ AAF34761	16/14; 62% identity (82% similar) with allergen Hev b 2, E,1e-7	11/1; 100% identity with putative allergen Cap a Glucanase, E = 0/ Sola t Glucanase, Hev b 2, Sola l Glucanase
Enolase [*C. annum*]/ XP_016542903	19/17; 90% identity (98% similar) with allergen Hev b 9	91/29; 89% identity with Amb a 12.0102, Hev b 9, Zea a 22, Cyn d 22.0101, E = 0/ Gas ac 2, Ano fi 2, Sal s 2, Tak ru 2, Ruda ni 2, Ict pu 2, Ory la 2, Dan re 2, Bos d Enolase, Gil mi 2, Ruda m 2, Pan h 2.0101 and Tet ni 2
Hypothetical protein BC332_07738 [*C. chinense*]/ PHU22631	72/12; 53% identity (83% similar) with putative allergen Ber e 2.0101, E < 1e-07	1 score; 71% identity with allergen Sin a 2, E = 015
Lichenase [C. annum]/ XP_016563240	15/14; 71% identity (90% similar) with allergen Hev b 2, E < 1e-7	31/11; 84% identity with putative allergen Cap a Glucanase, E < 1e-7/Sola t Glucanase, Hev b 2, Sola l Glucanase, Vit v Glucanase
NADPH-dependent aldehyde reductase 1, chloroplastic [*C. annuum*]/ XP_047250615	4/1; 75% identity (89% similar) with putative unassigned allergen of sesame (ACB55491.1), E < 1e-07	No matches
Oleosin 21.2 kDa [*C. annuum*]/ PHT73078	10/4; 62% identity (84% similar) with putative allergen Ses i 4.0101, E < 1e-07	1 score; 61% identity with putative allergen Ses i 4.0101, E < 1e-7
Osmotin-like protein OSML13 precursor [*C. annuum*]/ NP_001311827	35/15; 100% identity (100% similar) with putative allergen Cap a 1.0101, E < 1e-07	34/12; 100% identity with putative allergen Cap a 1.0101, E < 1e-7/Sola l TLP, Nic t Osmotin, Act d 2
Pathogenesis-related protein 10 [*C. annuum*]/ AAF63519	192/3; 73% identity (89% similar) with putative allergen Sola l 4.0201, E < 1e-07	10/6; 100% identity with putative allergen Cap a 4, E = 0.0/Cap ch 17kD, Sola l 4.0201
Pathogenesis-related protein 10 [C. chinense]/ CAI51309	193/3; 63% identity (88% similar) with putative allergen Sola l 4.0201, E < 1e-07	8/5; 100% identity with in silico generated allergen Cap ch 17kD, E < 1e‑7/Cap a 4, Sola l 4
Prunin 1 Pru du 6.0101 [*C. annuum*]/ XP_016570626	67/8; 56% identity (81% similar) with putative allergen Ses i 7.0101, E < 1e-07	1 score; 64% identity with putative allergen Fag e 1, E < 1e-7
Putative pathogenesis related protein [*C. chinense*]/ CAI48023	192/28; 79% identity (94% similar) with putative allergen Sola l 4.0201, E < 1e-07	10/5; 100% identity with in silico generated allergen Cap ch 17kD, E < 1e-7/Sola l 4.0201, Sola l 4
serpin-ZX-like [*C. annuum*]/ XP_016568712	8/2 scores; 53% identity (85% similar) with allergen Tri a 33.0101, E < 1e-07	No matches
Suberization-associated anionic peroxidase 2 [*C. annuum*]/ PHT90648	No matches	2/1; 83% identity with putative allergen Sola l Peroxidase, E < 1e-7
Vicilin Jug r 2.0101 [*C. annuum*]/ XP_016567997	60/0 scores; 42% identity (79% similar) with allergen Cor a 11.0101, E < 1e-07	No matches

^a^Results of protein analysis from Table 1 (the last column), ^b^Protein name [organism]/NCBI database accession, ^c^Allergenicity hazard of protein estimated from Allergen Online database (http://www.allergenonline.org) using the Full FASTA 36 search algorithm (E-value Cutoff = 1)): total scores/number of scores with > 50% identity and E < 1e-7; the allergenic sequence with the best score, ^d^Allergenicity hazard of protein estimated from Allergome database (http://www.allergome.org) using the NCBI blastp algorithm: total scores with % identity Cutoff = 60 and E-value Cutoff = 1/scores with 70% identity with E < 1e-7; the allergenic sequence with the best score/remaining allergenic sequences with min 70% identity and E < 1e-7.

Table 2 shows the allergenicity risk of proteins recognised by MASCOT as putative contaminants of the raw material, including proteins finally assigned to *Capsicum* based on BLAST matching. Three of them showed high homology (79–100% identity, E < 1e-7) to allergenic proteins with partially or fully documented IgE epitopes, including Cas s 9.0101, Hev b 1.0101 and Hev b 3.

#### Immunomodulatory potential of proteins

Proteins identified in silico as having a high risk of allergenicity, based on high amino acid sequence identity with allergenic proteins, were further screened in silico for the presence of proinflammatory epitopes (PiEs) and antibody-specific B cell epitopes (IgG, IgE and IgA), as well as for the presence of cytokine-inducing sequences (IL-4, IFN-γ and IL-6). The vast majority of them had peptides bearing potential PiEs or capable of inducing proinflammatory cytokines (Supplementary Table 2S). For PiEs, their possible amino acid sequences ranged from 23 to 123 for a window length of 15 and a threshold of 0.9, with the best scores ranging from 1.07 to 1.87. For IL-4 and IFN-γ, with a window length of 15 and a threshold of 0.7, it was 0–10 and 0–24 with best scores of 0.52–1.16 and 0.27–1.39 for IL-4 and IFN-γ, respectively. Possible sequences of IL-6 inducing peptides ranged from 8 to 107 and best scores from 0.18 to 0.61, with a window length of 15 and a threshold of 0.11 (upper value). The in silico analysis revealed that the proteins may differ in their ability to induce antibodies secretion. The number of Abs-inducing peptides was 0–78, 0–17 and 0–20 (best scores 0.92–1.70, 0.92–1.25 and 0.96–1.26), for IgG, IgE and IgA, respectively (Supplementary Table 2S). In turn, IgE epitopes, mapped with the AlgPred 2.0 tool, were present on 10 of all identified proteins (Supplementary Table 2S). The highest number of IgE epitopes was on rubber elongation factor protein, a contaminant of spices*.*

#### Immunomodulatory activity of selected peptides

High-risk allergenic proteins and their peptides with pro-inflammatory antigenic regions estimated by ProInflam (those with the highest SVM score and those containing IgE epitopes mapped by AlgPred 2) and peptide sequences with predicted IgE-specific B-cell epitopes predicted by IgPred (those with the highest score) were screened for binding to human major histocompatibility complex class II (MHC II), and the peptides themselves were additionally screened for cytokine induction. The PiE scores of all tested peptides were predominantly highly positive but ranged from − 0.04 (negative) to 1.89 (Table 4). The peptides were able to bind, on average, to 19 of the 24 HLA alleles tested (8.1 per 12 DRB1, 4.8/5 DQ and 6.5/7 DP), and whole proteins were able to bind on average to 23.7 of the 24 alleles tested (11.7/12 DRB1, 4.96/5 DQ and 7/7 DP). With the default settings of the tools used, approximately 24 and 26 of the 45 peptides examined appeared to be potential IL-4 and/or IL-10 inducers, whereas all appeared to be IFN-γ inducers. The inducer scores obtained were 0.22–1.55, 0.32–1.85 and 0.26–1.60 for IL-4, IL-10 and IFN-γ, respectively.

**Table 4:** Immunomodulatory activities of selected peptides bearing pro-inflammatory antigenic regions or IgE epitopes–results of in silico analysis.

Origin^a^	Peptide sequence^b^	Proinflammatory response/SVM score	Number of HLA alleles^c^			Cytokines^d^		
						IL-4	IL-10	IFN-γ
			DRB1	DQ	DP	Hybrid (SVM + motif based)	SVM based	Motif and SVM hybrid
11S globulin seed storage protein 2 [*C. baccatum*]/PHT52858	(1) EKKRTQIGLRKQSTQKFQNI (2) SQPSQ**RIESEG**GFTELWDEN (3) RIRQGDVVAIPAGAAHWCFN	Proinflammatory/1.47 Negative/0.66 Negative/0.16	9/12 7/12 8/12	5 5 5	7 6/7 7	Inducer/0.35 Inducer/1.40 Inducer/1.35	Inducer/1.85 Non-inducer/0.29 Non-inducer/ − 0.30	Inducer/1.26 Inducer/0.88 Inducer/1.43
11S globulin seed storage protein Ana o 2.010 [*C. annuum*]/ XP_016565958	(1) NFAIVKKAGDQGLEYIAFKT (2) HY**NNA**PQLIYIVQGRGLLGV	Proinflammatory/1.64 Proinflammatory/1.14	7/12 12	5 5	6/7 7	Inducer/0.29 Non-inducer/0.06	Inducer/0.81 Inducer/0.89	Inducer/1.06 Inducer/1.07
11S globulin seed storage protein Jug r 4-like [*C. annuum*]/ XP_016565474	(1) SGNVFSGFEQELLAEAFGVD (3) QQRFQQQQGQCQLNRLSPQE	Proinflammatory/1.66 Negative/ − 0.04	8/12 8/12	5 5	7 5/7	Non-inducer/0.10 Non-inducer/ − 1.13	Inducer/0.87 Non-inducer/ − 0.15	Inducer/0.94 Inducer/0.38
17.8 kDa class I heat shock protein [*C. annuum*]/ XP_016577734	(1) NSMFDPFAMDVFDPFRELGF	Proinflammatory/1.38	9/11	5	7	Non-inducer/-0.75	Inducer/0.32	Inducer/0.93
18.5 kDa class I heat shock protein [*C. baccatum*]/ PHT28807	(1) SNIFDPISLDLWDPFEGFPI	Proinflammatory/1.19	9/11	5	7	Non-inducer/ − 0.62	Inducer/0.55	Inducer/1.09
Actin-7-like [*C. annuum*]/ XP_016565383	(1) YNSIMKCDVDIRKDLYGNIV	Proinflammatory/1.80	12	5	7	Inducer/1.46	Non inducer/0.02	Inducer/0.74
Anionic peroxidase [*C. chinense*]/ CAI48071	(1) RGFEVIAQAKQSVVDTCPNI	Proinflammatory/1.28	9/11	5	6/7	Inducer/0.23	Inducer/0.88	Inducer/1.41
Basic 30 kDa Endochitinase precursor [*C. annuum*]/ NP_001311510	(1) TTGDTAVRKREIAAFFAQTS (2) APG**RKYFG**RGPIQISYNYNY	Proinflammatory/1.38 Proinflammatory/1.07	10/12 12	5 5	7 7	Inducer/0.93 Inducer/0.55	Inducer/1.10 Non-inducer/0.23	Inducer/1.14 Inducer/1.18
Basic beta-1,3-glucanase [*C. annuum*]/ AAS20585	(1) NIEVMLGVPNSIFKTLLPPF (2) RFLDIFAEN**NNA**TSTFFKSD	Proinflammatory/1.44 Proinflammatory/1.01	8/12 7/12	5 5	7 7	Non-inducer/ − 0.95 Inducer/1.40	Inducer/0.71 Non-inducer/0.21	Inducer/1.60 Inducer/0.90
Chloroplast small heat shock protein class I [*C. frutescens*]/ AAQ19680	(1) SNIFDPVSLDLWDPFEGFPI (3) VDVPGIKREEVKVQVEEGRI	Proinflammatory/1.17 Negative/ 0.20	9/11 8/11	5 5	7 7	Non-inducer/-0.66 Inducer/1.16	Inducer/0.32 Non-inducer/ − 0.12	Inducer/1.02 Inducer/1.10
Enolase [C. annum]/ XP_016542903	(1) AVRNVPLYKHIADLAGNKKL	Proinflammatory/1.43	6/12	5	7	Non inducer/ − 1.17	Non inducer/-0.31	Inducer/0.82
Hypothetical protein BC332_07738 [*C. chinense*]/ PHU22631	(1) SGNVFSGFEQELLAEAFGVD (3) IANSYQISREEARRLKFNREE	Proinflammatory/1.66 Negative/ 0.44	8/12 9/12	5 5	7 7	Non-inducer/0.10 Inducer/1.14	Inducer/0.87 Inducer/1.34	Inducer/0.94 Inducer/1.01
Lichenase [*C. annum*]/ XP_016563240	(1/2) LGVPNSDLQNIAAN**PSN**ANS (2) RFLDISAEN**NNA**TSTSLKSD (2) ATT**NNA**ATYYRNLIQHVRRG	Proinflammatory/1.34 Proinflammatory/0.86 Negative/0.68	9/12 2/12 8/12	5 3/5 5	7 3/7 7	Non-inducer/– 0.10 Inducer/0.44 Non-inducer/ − 0.09	Inducer/0.39 Inducer/0.46 Non-inducer/ − 0.20	Inducer/1.06 Inducer/1.37 Inducer/1.17
NADPH-dependent aldehyde reductase 1, chloroplastic [*C. annuum*]/ XP_047250615	(1) AFTYVKSQEEKDAQDTLKLL (2) DILV**NNA**AEQYEASSVEEIN	Proinflammatory/1.73 Negative/0.53	3/10 7/10	5 5	7 5/7	Non-inducer/ − 0.30 Inducer/0.22	Inducer/1.04 Non-inducer/0.03	Inducer/1.03 Inducer/0.89
Oleosin 21.2 kDa [*C. annuum*]/ PHT73078	(1) QAIQSKAQEGKESARTDVRT	Proinflammatory/1.15	6/12	5	5/7	Inducer/0.30	Non-inducer/ − 0.21	Inducer/1.38
Osmotin-like protein OSML13 precursor [*C. annuum*]/ NP_001311827	(1) KFFKKRCPDAYSYPQDDATS	Proinflammatory/1.21	8/12	5	7	Inducer/1.30	Inducer/0.87	Inducer/0.42
Pathogenesis-related protein 10 [*C. annuum*]/ AAF63519	(1) HNVHKEKANDLLKAIEAYLL (2) NNLVSKLAPDVKSI**ENVEG**D	Proinflammatory/1.34 Proinflammatory/1.02	7/12 11/12	5 5	7 7	Non-inducer/ − 1.06 Inducer/0.40	Inducer/0.76 Non-inducer/ − 0.46	Inducer/0.27 Inducer/0.26
Pathogenesis-related protein 10 [C. annuum]/ CAI51309	(1) TKYSLIEGDALANKADSVDY	Proinflammatory/1.30	8/12	5	7	Inducer/1.55	Non-inducer/0.04	Inducer/0.66
Prunin 1 Pru du 6.0101 [*C. annuum*]/ XP_016570626	(1) GFDAQLLSEAFNVDFEMIRK (2) SLIDTS**NNA**NQLDLTFRKFF (2) YNPRGGR**IAT**ANSNTLPVLN	Proinflammatory/1.66 Negative/0.62 Proinflammatory/1.33	9/12 2/12 10/12	5 5 3	7 7 7	Inducer/1.15 Inducer/1.44 Non-inducer/ − 0.15	Inducer/0.69 Non-inducer/0.23 Inducer/0.74	Inducer/0.72 Inducer/0.49 Inducer/0.80
Putative pathogenesis related protein [*C. chinense*]/ CAI48023	(1) HNVGKEKAIDLLKAVEAYLL (3) FVEGGPIKYLKHKIHVVDEK	Proinflammatory/1.57 Negative/0.62	11/12 11/12	5 5	7 7	Non-inducer/ − 1.32 Inducer/0.29	Inducer/1.06 Non-inducer/ − 0.17	Inducer/1.49 Inducer/1.32
Rubber elongation factor protein [*H. brasiliensis*]/ P15252	(1) GQGEGLKYLGFVQDAATYAV (2) **DRSLPPIVKDASIQVVSAIR** (3) FSNVYLFAKDKSGPLQPGVD	Proinflammatory/1.48 Proinflammatory/0.82 Negative/0.43	7/12 9/12 7/12	5 5 4/5	7 7 6/7	Non-inducer/0.15 Non-inducer/0.16 Non-inducer/0.06	Non-inducer/0.12 Non-inducer/ − 0.04 Inducer/0.64	Inducer/1.24 Inducer/0.93 Inducer/0.93
Serpin-ZX-like [*C. annuum*]/ XP_016568712	(1) QTLPLKHSFKQIVDNVYKAA (3) AGVVKLRALMVDEKVDFVAD	Proinflammatory/1.89 Proinflammatory/0.82	8/12 11/12	5 5	7 7	Non-inducer/0.17 Inducer/0.29	Inducer/1.62 Non-inducer/ − 0.38	Inducer/0.84 Inducer/1.35
Stress-related protein [*C. baccatum*]/ PHT38668	(1) EPTAKDLYAKYEPIAEKNAV	Proinflammatory/1.66	8/9	5	7	Inducer/1.12	Non-inducer/ − 0.74	Inducer/1.14
Suberization-associated anionic peroxidase 2 [*C. annuum*]/ PHT90648	(1) RGFEVIAQAKQSVVDTCPNI	Proinflammatory/1.28	9/11	5	6/7	Inducer/0.23	Inducer/0.88	Inducer/1.41
Vicilin Jug r 2.0101 [*C. annuum*]/ XP_016567997	(1) ATGDSNLRMVGFGINGHNSR (2) HQRQGH**KVVR**GCLSVGDFFV (3) QGIVIKASEEQIRAISQHAS	Proinflammatory/1.79 Proinflammatory/0.88 Proinflammatory/0.73	8/12 5/12 8/12	5 5 5	6/7 7 7	Non-inducer/ − 0.44 Non-inducer/ − 0.58 Inducer/0.66	Inducer/0.70 Inducer/0.53 Inducer/0.53	Inducer/0.67 Inducer/0.90 Inducer/1.46

^a^Protein name [organism]/NCBI database accession, ^b^Peptides obtained with the ProInflam (http://metagenomics.iiserb.ac.in/proinflam/) or IgPred (https://webs.iiitd.edu.in/raghava/igpred/) web servers at default settings; (1) peptide with the highest proinflammatory score, (2) peptide bearing IgE epitope (highlighted in bold) mapped with AlgPred 2.0 web server (https://webs.iiitd.edu.in/raghava/algpred/), and (3) the sequence with predicted IgE-specific B-cell epitope obtained with IgPred web server (with the highest score). The underline regions correspond to the number of HLA-DRB1 alleles specified for the sequence (http://www.ddg-pharmfac.net/EpiTOP3/). ^c^Number of HLA alleles reacting with peptide as binders (http://www.ddg-pharmfac.net/EpiTOP3/): number of alleles reacting with the indicated peptide/number of alleles reacting with peptides in whole protein. ^d^Cytokine inducing ability of the indicated peptide, estimated for IL-4 (https://webs.iiitd.edu.in/raghava/il4pred/), IL-10 (https://webs.iiitd.edu.in/raghava/il10pred/) and IFN-γ (https://webs.iiitd.edu.in/raghava/ifnepitope/) at default settings: action/ score.

## Discussion

Our studies using the immunoblotting technique showed the presence of IgE antibodies to paprika proteins in the serum of patients whose medical tests for these allergens were inconclusive, but the likely cause of the allergic reaction was the ingestion of *Capsicum* spices, or cross-reactivity with allergens of similar epitope structure. The IgE reactive proteins appeared to be approximately 50, 33 and 20 kDa. A total of 53 proteins were identified in the immunoreactive bonds, five of which showed 100% identity to *Capsicum* allergens described in the *Allergome* database: Cap a Glucanase, Cap a 1.0101, the in silico generated Cap ch 17kD and Cap a 4. The IgE reactive protein could also be lichenase, which showed 84% identity with Cap a Glucanase (E < 1e-7). According to the *Allergome* database, the allergenicity scores for these putative *Capsicum* allergens were based on IgE immunoblotting tests. Among the paprika allergens registered in the WHO/IUIS database, Cap a 1 (osmotin- thaumatin-like protein) was found in the 20 kDa band. As for the other two allergens, profilin (Cap a 2) and gibberellin-regulated protein (Cap a 7), although they are soluble, our sera did not react positively with proteins with a molecular weight of less than 15 kDa, so they did not react with Cap a 2 and Cap a 7 allergens. Of the remaining proteins identified in the IgE reactive bands, 11S globulin seed storage protein Jug r 4-like, 17.8 kDa and 18.5 kDa class I heat shock proteins, actin-7-like, anionic peroxidase, basic 30 kDa endochitinase precursor, enolase, hypothetical protein BC332_07738, NADPH-dependent aldehyde reductase 1, chloroplastic, and suberization-associated anionic peroxidase 2 showed very high identity (≥ 70; E < 1e-7) to known allergens or putative allergens from other plants used in the food industry. Alignment with such identity scores indicates a potential for allergenic cross-reactions. Cross-reactivity is unlikely for proteins with less than 50% identity to the entire protein sequence and is quite common above 70% identity^21^. According to the authors of the *AllergenOnline* database, sequences of two proteins having published evidence of cross-reactivity will align in *AllergenOnline.org* with a high percent identity (> 50% over nearly full length) and have an E score (statistical expectation score) of less than 1e-7 (0.0000001)^22^. Our in silico analysis showed that cross-reaction of paprika proteins with latex (Hev b 2, Hev b 9, Hev b 11), tomato (Sola t Glucanase, Sola l Glucanase, Sola I TLP, Sola I Peroxidase, Sola I 4), tobacco (Nic t Osmotin), grapes (Vit v Glucanase), mustard (Sin a 2), kiwi (Act d 2), sesame (Ses i 5, unassigned sesamum seed maturation-like protein group; ACB55491.1), avocado (Pers a 1), wheat (Tri a Endochitinase), maize (Zea a 22), banana (Mus xp 2), chestnut (Cas s 9), hazel (Cor a 13), molds (Asp f gamma Action), meadow plants (Amb a 12, Cyn d 22), cattle (Bos d Enolase), crab (Chi o alpha) and even to mostly in silico generated fish allergens (Sal s alpha Actin; Gas ac 2, Ano fi 2, Sal s 2, Tak ru 2, Ruda ni 2, Ict pu 2, Ory la 2, Dan re 2, Gil mi 2, Ruda m 2, Pan h 2, Tet ni 2) are particularly like. Unfortunately, apart from negative results for tomato and potato, we have no confirmed information on whether our patients were hypersensitive to these proteins, so we cannot indicate what cross-reactions, if any, occurred. Reactivity of plant proteins with animal allergens seems unlikely at present, as it has not yet been clearly described. However, cross-reactivity of pollen with food proteins is increasingly observed. A dangerous allergen in peppers seems to be Cap a Glucanase, which has a high identity with the latex allergen Hev b 2. The same is true for enolase, which shows 90% identity with Hev b 9. MS analysis showed that spices were contaminated with latex. This could have exacerbated the allergic reaction through cross-reactivity between allergens. Unfortunately, we have no information whether our patients were allergic to latex. Latex has many recognised IgE epitopes, that can cross-react with many food proteins. Cases of paprika allergy associated with a latex-fruit syndrome have been reported, and seems to be quite common^23,24^. Nevertheless, Estrada-Rodriguez et al*.*^25^ described a case of paprika allergy in which they excluded latex and rubber allergy. Although the specific IgE was low (0.34 kU/L), they, like us, confirmed the presence of IgE-reactive proteins by immunoblotting technique. Palomares et al.^26^ detected reactive IgG and IgE peptide epitopes common to 1,3-beta-glucanase (Ole e 9) in extracts of ash and birch pollen, tomato, potato, banana, latex and paprika. However, the described latex food allergy syndrome is most commonly recognized in patients with hypersensitivity to latex, banana, kiwi, avocado, tomato, potato, chestnut, and peach^27^.

Allergic diseases can lead to eating disorders, psychosocial disadvantages, and inflammatory autoimmune diseases^28,29^. We tested the proinflammatory potential of proteins with high allergenic risk by in silico mapping some inflammatory and IL-4, IFN-γ and IL-6 inducing peptides, as well as those with IgG-, IgE- and IgA-specific B cell epitopes. The storage and defence proteins seem to stand out in terms of bioactivity tested. The storage protein prunin 1 Pru du 6.0101 showed the highest scores for the proinflammatory markers tested. Although it does not appear to have IgE-specific B cell epitopes, it does have short IgE epitopes, identified using AlgPred 2.0 software. This protein and the 11S globulin seed storage protein Ana o 2.0101 and 11S globulin seed storage protein Jug r 4-like showed the highest ability to induce IL-6, a proinflammatory cytokine that stimulates acute phase responses, haematopoiesis, and specific immune responses. In terms of IgE-specific B cell epitopes, 36% of the proteins with a high risk of allergenicity showed their presence, but the most remarkable results we observed for the hypothetical protein BC332_07738, serpin-ZX-like, 11S globulin seed storage protein Jug r 4-like, 11S globulin seed storage protein Ana o 2.0101 and rubber elongation factor protein. They have not yet been reported as potential paprika allergens, although they are likely to influence allergic reactions in sensitised individuals, which should be investigated.

The affinity of dietary peptides for MHC II is crucial in the development of allergy. The most common HLA (human MHC) alleles corresponding to MHC class II are HLA-DRB1 (12), HLA-DQ (5), and HLA-PD (7). HLA molecules act as receptors that bind lysosomal processed antigens and present them to T lymphocytes. This initiates an immune response; the production of cytokines, antigen-specific antibodies by B lymphocytes, and the formation of cytotoxic lymphocytes. Depending on the cytokines secreted, CD4^+^ T (T helper) cells polarise into Th1, Th2, Th17 or iTregs populations^30^. The paprika proteins studied carry peptides capable of inducing antibodies and proinflammatory cytokines, such as IL-4 or IL-6. IL-4 plays a key role in antibody isotype switching, stimulating IgE production, haematopoiesis and inflammation, and the development of appropriate effector T cell responses^31^. Its secretion is a characteristic Th2 cell response, resulting from the maturation of Th0 lymphocytes in the presence of IL-4 produced by previously activated Th2 cells, mast cells, basophils and NKT cells (Natural Killer T cells)^31^. Proteochemometric analysis using the EpiTOP3 tool revealed a high capacity of the tested proteins to bind to HLA alleles. We found the presence of numerous peptides that can be bound by the HLA-DRB1,—DQ and -DP alleles found on antigen-presenting cells. Twenty three of the 45 peptides examined appeared to be IL-4 inducers, including 11 with SVM-motif-based scores above 1. Six of them (derived from: actin-7-like, basic beta-1,3-glucanase, osmotin-like protein (Cap a 1 allergen), pathogenesis-related protein 10, prunin 1 Pru du 6.0101 and stress related protein) showed strong proinflammatory features, indicating a high probability of allergic reaction to their parent proteins, especially since, except for two derived from prunin 1 Pru du 6.0101 and osmotin-like protein, the peptides did not induce IL-10. IL-10 plays a crucial role in the development of tolerance by suppressing inflammation, altering the profile of activated effector cells, increasing the expression of tight junction proteins in the mucosa, and increasing the number of goblet cells^32^. Stress related protein significantly induced IFN-γ. The generation of IFN-γ by MHC class II activated CD4^+^ Th cells is important in the context of the accompanying proinflammatory response, but also plays an important role, depending on the allergen dose, in immune suppression and the induction of tolerance to allergenic proteins^31,33^. Only a reduction of the secretion of proinflammatory cytokines, e.g. IL-4 and IFN-γ, while increasing the levels of regulatory cytokines, such as IL-10, in the context of peptide potential discrimination, offers hope for a more targeted immunotherapy^34,35^. Of the above-mentioned highly immunoreactive proteins capable of inducing IgE, only basic beta-1,3-glucanase and pathogenesis-related protein 10 have been reported as potential paprika allergens (Cap ch 17kD and Cap a 4).

## Conclusions

The study showed that *Capsicum* spices possess many highly immunoreactive allergenic proteins/peptides, the presence of which can stimulate potent inflammatory mechanisms. Basic beta-1,3-glucanase (Cap a Glucanase), osmotin-like protein (Cap a 1.0101), pathogenesis related proteins 10 (Cap a 4, Cap ch 17kD) and putative pathogenesis related proteins (Cap ch 17kD) have already been reported as allergens or putative paprika allergens. However, other proteins may also be highly allergenic , such as 11S globulin seed storage protein Ana o 2.0101, 11S globulin seed storage protein Jug r 4-like, actin-7-like, hypothetical protein BC322_07738, lichenase, prunin 1 Pru du 6.0101, serpin-ZX-like, stress related protein or vicilin Jug r 2.0101 showing strong proinflammatory features. In addition, cross-reactivity of paprika proteins with latex (possible paprika contaminant), tomato, tobacco, grapes, mustard, kiwi, sesame, avocado, wheat, maize, banana, chestnut, hazel, molds, meadow plants, and even cattle, crab and fishes is possible and should be taken into account in allergy diagnosis, especially in the cases of idiopathic and non-IgE-mediated anaphylaxis, without exceeding norms of specific IgE antibodies.

## Materials and methods

### Protein extracts

Commercially available peppers spices (mild, spicy and chili) were used in the study. Proteins were extracted in (a) 10 mmol/L PBS (pH 7.0) containing 2% (w/v) polyvinylpolypyrrolidone (PVPP), 2 mmol/L ethylenediaminetetraacetic acid (EDTA), 10 mmol/L sodium diethyldithiocarbamate (DIECA) and 3 mmol/L sodium azide^36^, and in (b) 20 mmol/L Tris/HCL buffer (pH 7.4) containing 150 mM NaCl, 0.05% Tween 20, 1% sodium dodecyl sulfate (SDS) and 7% 2-mercaptoethanol (ME)^37^ by overnight shaking at 4 °C. After centrifugation at 12000×*g* for 60 min at 4 °C, the supernatants were collected and further centrifuged in Amicon Ultra centrifugal 3K devices (Merck Millipore Ltd., Cork, IRL) at 5000×*g* for 20 min. The concentrated extracts were collected, and aliquots were stored at − 20 °C for analysis. Protein content was determined by the Bradford method.

### Serum

Human sera were selected from the bank of sera collected at the IAR&FR PAS in Olsztyn between 2010 and 2014^38^. All procedures were approved by the Bioethics Committee of the Faculty of Medical Sciences of the University of Warmia and Mazury in Olsztyn (decision No. 2/2010 and 2/2016) and were performed in accordance with the standards of the Helsinki Declaration. Written informed consent was obtained from all subjects. The sera tested (3) were from patients (aged 32–57 years, female) with severe allergic reactions, presumably to paprika, including one episode of anaphylaxis (see Supplementary Table 3S). The EUROLINE Atopy Screen Panel normally used to diagnose the sera (EUROIMMUN AG, Lübeck, Germany) did not include paprika, so the sera were analysed using the Allercoat™ 6-ELISA and the Allergy Profile Pollen-Food Cross Reactions test (EUROIMMUN AG, Lübeck, Germany). Paprika-specific serum IgE levels were < 0.35 kU/L. Potato- and tomato-specific IgE antibodies were also not elevated. Sera were pooled due to similar clinical findings and the intended immunoblotting analysis.

### SDS-PAGE analysis

Extracted proteins (20 μg) were separated in the 12.5% polyacrylamide gel in the presence of the Tris–glycine buffer (192 mmol/L glycine, 25 mmol/L Tris and 0.1% SDS, pH 8.3; according to Laemmli^39^, using 4μL of Odyssey® Protein Molecular Weight Marker (10–250 kDa) (Li-COR Biotechnology, Lincoln, NE, USA). Electrophoresis was performed in a Mini PROTEAN 3 Cell apparatus (Bio-Rad Laboratories, Hercules, CA, USA) at 140V for 75 min. Gels were stained with a 0.1% solution of Coomassie Brilliant Blue R-250. Bands were detected on the ChemiDoc Imaging System (Bio-Rad Laboratories) and analysed using Image Lab software (Bio-Rad Laboratories) including densitometric analysis.

### Immunoblotting for IgE binding assay

Proteins were transferred onto nitrocellulose membranes (Sigma-Aldrich, St. Louis, MO, USA) by wet electrotransfer in a buffer of Tris–glycine (pH 8.3) with methanol (192 mmol/L glycine, 25 mmol/L Tris and 20% (v/v) methanol) according to Towbin et al.^40^ at 25 mA for 20 h. Membranes were washed in PBS (pH 7.4) for 5 min at room temperature (RT), then blocked in the Odyssey® blocking buffer (Li-COR Biotechnology), pH 7.2–7.6, for 2 h at RT according to Markiewicz et al.^41^ and incubated overnight at 4 °C in a solution of human sera diluted twice in blocking buffer containing 0.1% Tween 20. The membranes were then rinsed four times in the PBS-T buffer (PBS, pH 7.4, containing 20% Tween 20). Detection of human IgE reactive proteins was performed by incubating the membranes for 90 min at RT in a solution containing mouse monoclonal anti-human IgE antibodies (Sigma-Aldrich) labelled with IRDye® 800CW (Li-COR Biotechnology). Anti-human IgE secondary antibodies were diluted 1:500 with Odyssey® blocking buffer (pH 7.2–7.6) containing 0.1% Tween 20 and 0.01% SDS. Signal detection was performed using the ChemiDoc Imaging System (Bio-Rad Laboratories) and analysed using Image Lab software (Bio-Rad Laboratories).

### Identification of proteins by LC–MS/MS analysis

Bands identified as IgE reactive were excised from the gel, destained in 50 mM NH_4_HCO_3_ solution in 50% ACN, reduced with 10 mM DTT in 100 mM NH_4_HCO_3_ and alkylated with 50 mM iodoacetamide solution in 100 mM NH_4_HCO_3_. Proteins were then identified by mass spectrometry (MS) after in-gel digestion with 10 ng/mL trypsin (Promega, Madison, WI, USA) overnight at 37 °C. Trifluoroacetic acid was added to a final concentration of 0.1% to stop digestion. MS analysis was performed by LC–MS/MS technique in the Laboratory of Mass Spectrometry (IBB PAS, Warsaw) using a nanoACQUITY UPLC system (Waters Corporation, Milford, MA, USA) coupled to an LTQ-Orbitrap Velos mass spectrometer (Thermo Fisher Scientific, Waltham, MA, USA). The sample was applied to the nanoACQUITY UPLC trapping column (Waters Corporation, Milford, MA, USA) using water containing 0.1% formic acid as the mobile phase. The peptide mixture was then transferred to the nanoACQUITY UPLC BEH C18 column (Waters Corporation, 75 µm inner diameter, 250 mm long) and a CAN gradient (5–35% over 180 min) was applied in the presence of 0.1% formic acid at a flow rate of 250 nL/min. Eluted peptides were electrosprayed directly into the mass spectrometer operating in positive ion mode at a voltage of 2 kV. Spectra were recorded in full MS mode in profile mode at 60,000 resolution with a scan range of 400–2000 m/z. Each sample was washed three times prior to measurement to avoid cross-contamination and the final MS wash was checked for cleanliness. Raw data were searched using MASCOT (Matrix Science Ltd., London, UK) against the SwissProt database—taxa Green Plants (Viridiplantae), but also against all entries. Search parameters were: enzyme, trypsin; peptide mass tolerance, 20 ppm; fragment ion tolerance, 0.1 Da; fixed modifications, carbamidomethyl (C); variable modifications, oxidation (M). For each identified protein, the significance threshold of *p* < 0.05, the ions score or expected cut-off-43 and the highest emPAI value were considered significant. Finally, the identification results were checked using the Basic Local Alignment Search Tool (BLAST) against *Capsicum* taxid (https://www.ncbi.nlm.nih.gov). Proteins indicated by BLAST with the peptides on which the identification by MASCOT was based were found to be derived from *Capsicum.*

### In silico analysis of proteins and peptides

Protein allergenicity and proinflammatory activity were investigated using online tools. The in silico protein sequence analysis used was partially described by Ogrodowczyk et al.^42^.

#### Recognition of protein allergenicity

Sequences of proteins identified by MS analysis and BLAST were retrieved from NCBI and used for in silico allergenicity analyses. Proteins with sensitising potential were selected based on sequences deposited in the Allergome (https://www.allergome.org) and AllergenOnline v. 21 (FARRP; http://www.allergenonline.org) databases. Questionable results were further checked using the Allermatch database (http://allermatch.org). The allergenic potential of the protein was estimated using the full-length alignment and, in the absence of positive results, using 8- or 6-amino acid exact match methods. Prediction of IgE epitopes was performed using the AlgPred 2.0 server (https://webs.iiitd.edu.in/raghava/algpred2/)^43^.

#### Screening for proinflammatory activity of proteins with high risk of allergenicity

The ProInflam web server (http://metagenomics.iiserb.ac.in/proinflam) was used to predict antigenic regions that induce a proinflammatory response, the IL4pred tool (https://webs.iiitd.edu.in/raghava/il4pred/) to map IL-4 inducing peptides, while IFNepitope (https://webs.iiitd.edu.in/raghava/ifnepitope/) and IL-6Pred (https://webs.iiitd.edu.in/raghava/il6pred/) were used to map INF-γ and IL-6 inducing peptides, respectively^44–46^. The IgPred web server (https://webs.iiitd.edu.in/raghava/igpred/) was used to predict protein IgG, IgE and IgA specific B cell epitopes^47^.

#### Prediction of peptide-MHC II binding

The high-risk allergenic proteins and their peptides with proinflammatory antigenic regions estimated by ProInflam (the one with the highest SVM score and those containing IgE epitopes mapped by AlgPred 2) and peptide sequences with predicted IgE-specific B-cell epitopes predicted by IgPred (those with the highest score) were screened for binding to human major histocompatibility complex class II (MHC II). Protein sequences and peptides were uploaded to the EpiTOP3 server (http://www.ddg-pharmfac.net/EpiTOP3/), which is designated to predict binding to human leukocyte antigen (HLA) alleles corresponding to MHC class II using proteochemometric models. An IC50 threshold of 6.3 for peptide/HLA complexes was used for analysis^48,49^.

#### Prediction of peptide/MHC II complexes inducing IL-4, IL-10 and IFN-γ

Peptide sequences analysed as above were further screened for their ability to induce IL-4, IL-10 and IFN-γ. The IL4pred (http://crdd.osdd.net/raghava/il4pred/), IL-10pred (http://crdd.osdd.net/raghava/IL-10pred/) and IFNepitope (https://webs.iiitd.edu.in/raghava/ifnepitope/) tools were used for analysis with default settings^31,44,50^.

### Statistical analysis

Statistical parameters used in analyses requiring specialised software linked to an instrument/tool are described in the analytical method/online tool used and were briefly summarized by Ogrodowczyk et al.^42^. Densitometric data were expressed as mean ± SD from three independent assays. Student’s *t* test was used to compare isolation methods, while one-way ANOVA followed by post hoc Duncan or Kruskal–Wallis tests were used to compare protein isolates in the tested spices. Calculations were performed using *Statistica v. 13* (Statsoft, Kraków, Poland). Differences were considered significant at *p* < 0.05.

All procedures and methods were performed in accordance with relevant guidelines and regulations.

### Supplementary Information

Supplementary Information.

## Data Availability

The mass spectrometry proteomics data were deposited at the ProteomeXchange Consortium via the PRIDE partner repository^51^ under the accession numbers PXD039651 and 10.6019/PXD039651.
