# Supplementary material for: Immunoreactive proteins of Capsicum-based spices as a threat to human health: mass spectrometry analysis and in silico mapping
Source: Sci Rep. 2023 Oct 18;13:17723. doi: 10.1038/s41598-023-44775-3 (PMC10584839; doi:10.1038/s41598-023-44775-3)
Supplement: Supplementary file 1 — Supplementary Information. [file 41598_2023_44775_MOESM1_ESM.docx]

# **Supplementary Material**

# Supplementary Table 1S. Allergenicity hazard of *Capsicum* proteins – remaining results of *in silico* analysis.^a^

| **Protein^b^** | **AllergenOnline scores^c^** | **Allergome scores^d^** | **Allermatch^e^** |
| --- | --- | --- | --- |
| 17.3 kDa class II heat shock protein [*C. annuum*]/ XP_016563710 | 4/0/0 scores; 31% identity (70% similar) with putative allergen Ana br 1, E=0.065 | no matches | 46% identity (72% similar) with putative allergen Cas s 9.0101, E<1e-07 |
| 22.0 kDa class IV heat shock protein [*C. chinense*]/ PHT97509 | 7/0/0 scores; 26% identity (61% similar) with putative allergen Spi sa 1, E=0.0073 | no matches | 45% identity (73% similar) with putative allergen Cas s 9.0101, E<1e-07 |
| 2-Cys peroxiredoxin BAS1, chloroplastic [*C. annuum*]/ XP_016543590 | 4/2/0 scores; 34% identity (65% similar) with allergen Tri a 32, E<1e-07 | no matches | the same as AllergenOnline |
| annexin [*C. annuum*]/ CAA63710 | 2/0/0 scores; 27% identity (55% similar) with putative allergen Ven ph 1, E=0.65 | no matches | 27% identity (56% similar) with unproven allergen Bal r 1, E=0.65 |
| aspartyl protease AED3 [*C. annuum*]/ XP_016557272 | 3/0; 38% identity (69% similar) with unassigned pollen allergen (BAJ04354), E<1e-7 | 1/0; 70% identity with putative allergen Lac s AP, E=0.16 | 34% identity (70% similar) with putative allergen Cry j AP, E<1e-7 |
| desiccation-related protein PCC13-62 [*C. annuum*]/ KAF3614133 | no matches | no matches | no matches |
| fibrillin [*C. annuum*]/ CAA50750 | no matches | no matches | no matches |
| GDSL esterase/lipase At1g71250 [*C. annuum*]/ XP_016563440 | 4/0 scores; 24% identity (53% similar) with putative allergen Hev b 13.0101, E<1e-07 | no matches | the same as AllergenOnline |
| glutathione S-transferase [*C. annuum*]/ AAN39918 | 14/0; 25% identity (62% similar) with allergen Per a 5, E<1e-7 | no matches | the same as AllergenOnline |
| putative chromoplastic oxydo-reductase [*C. annuum*]/ CAA54961 | no matches | no matches | 26% identity (51% similar) with putative allergen Mala s 12, E=1.3 |
| hypersensitive response assisting protein [*C. annuum*]/ AAD50436 | 4/2/0 scores; 24% identity (52% similar) with putative allergen Cry j chitinase, E<1e-07 | no matches | the same as AllergenOnline |
| late embryogenesis abundant protein D-29 [*C. annuum*]/ XP_016551859 | 19/0 scores; 28% identity (56% similar) with allergen Gly m 7, E=0.024 | no matches | the same as AllergenOnline |
| Late embryogenesis abundant protein D-34 [*C. chinense*]/ PHU21880 | no matches | no matches | no matches |
| lipocalin protein [*C. annuum*]/AAT71313 | 9/3/0 scores; 24% identity (53% similar) with putative allergen Per a 11.0101, E=1.9e-5 | no matches | the same as AllergenOnline |
| PGIP [*C. annuum*]/ ACB30360 | 2/1/0 scores; 34% identity (71% similar) with putative allergen Tri a 23kd, E<1e-07 | no matches | the same as AllergenOnline |
| phosphoglycerate kinase, chloroplastic [*C. annuum*]/ PHT77520 | no matches | no matches | 25% identity (67% similar) with putative allergen Mer p 1, E=3.2 |
| Serine carboxypeptidase 3 [*C. chinense*]/ PHU03819 | 7/0 scores; 30% identity (60% similar) with putative unassigned allergen (P08819), E<1e-07 | no matches | 37% identity (62% similar) with putative allergen Api m 9, E<1e-07 |
| small heat shock protein [*C. annuum*]/ AAP57477 | no matches | no matches | 35% identity (69% similar) with putative allergen Cas s 9.0101, E<1e-07 |
| small heat shock protein, chloroplastic [*C. annuum*]/ NP_001311883 | no matches | no matches | 36% identity (65% similar) with putative allergen Cas s 9.0101, E<1e-07 |
| stress-related protein [C. baccatum]/ PHT38668 | 2/0 scores; 46% identity (74% similar) with allergen Hev b 3, E<1e-7 | no matches | 46% identity (74% similar) with allergen Hev b3, E<1e-7 |
| thioredoxin peroxidase [*C. annuum*]/ AAL35363 | 10/8/0 scores; 38% identity (67% similar) with putative allergen Mala s 5.0101, E<1e-07 | no matches | the same as AllergenOnline |
| Transaldolase [*C. chinense*]/ PHT54042 | 3/0 scores; 33% identity (63% similar) with putative allergen Pen ch 35.0101, E=0.2 | no matches | the same as AllergenOnline |
| Xyloglucan endotransglucosylase/ hydrolase protein 31-like [*C. annuum*]/ XP_016547781 | 4/1/0 scores; 28% identity (53% similar) with allergen Asp f 9.0101, E<1e-07 | no matches | 28% identity (53% similar) with allergen Tri a 26, E=0.58 |

^a^Results of protein analysis from Table 1 (the last column). ^b^Protein name [organism]/NCBI database accession. ^c^Allergenicity hazard of protein estimated from Allergen Online database (http://www.allergenonline.org) using the Full FASTA 36 search algorithm (E-value Cutoff=1)): total scores/number of scores with E<1e-4/number of scores with >50% identity and E<1e-7; the allergenic sequence with the best score. ^d^Allergenicity hazard of protein estimated from Allergome database (http://www.allergome.org) using the NCBI blastp algorithm (% identity Cutoff=60): total scores with E-value Cutoff=1/including scores with 70% identity; the allergenic sequence with the best score. ^e^Allergenicity hazard of protein estimated from Allermatch database (http://www.allermuch.org) using a full FASTA alignment and AllergenDB original sequences (E-value Cutoff=1): the allergenic sequence with the best score.

Supplementary Table 2S. Immunomodulatory activity of proteins with high allergenicity hazard – results of *in silico* analysis.

| Protein^a^ | IgE epitope^b^ [SEQUENCE] | Pro-inflamatory  epitopes^c^ [PiEs] | Cytokine inducing peptides^d^ | | | Antybody-specific B-cell epitopes^e^ | | |
| --- | --- | --- | --- | --- | --- | --- | --- | --- |
|  |  |  | IL-4 | IFN-γ | IL-6 | IgG | IgE | IgA |
| 11S globulin seed storage protein 2 [*C. baccatum*]/ PHT52858 | [RIESEG] | 80/1.47 | 3/0.82 | 4/0.95 | 75/0.37 | 21/1.47 | 4/0.99 | 18/1.11 |
| 11S globulin seed storage protein Ana o 2.0101 [*C. annuum*]/ XP_016565958 | [NNA] | 113/1.07 | 1/0.78 | 14/1.19 | 107/0.54 | 20/1.18 | 7.1.25 | 7/1.07 |
| 11S globulin seed storage protein Jug r 4-like [*C. annuum*]/ XP_016565474 |  | 116/1.52 | 0/0.67 | 24/1.25 | 97/0.36 | 13/1.05 | 17/1.16 | 2/0.96 |
| 17.8 kDa class I heat shock protein [*C. annuum*]/ XP_016577734 |  | 47/1.48 | 1/0.70 | 0/0.60 | 15/0.28 | 37/1.52 | 0 | 0 |
| 18.5 kDa class I heat shock protein [*C. baccatum*]/ PHT28807 |  | 28/1.53 | 1/0.89 | 0/041 | 17/0.36 | 1/0.92 | 0 | 0 |
| actin-7-like [*C. annuum*]/ XP_016565383 |  | 103//1.84 | 0/0.58 | 7/1.00 | 19/0.29 | 45/1.38 | 0 | 14/1.07 |
| anionic peroxidase [*C. chinense*]/ CAI48071 |  | 46/1.32 | 5/0.89 | 2/0.77 | 27/0.38 | 33/1.17 | 0 | 0 |
| basic 30 kDa endochitinase precursor [*C. annuum*]/ NP_001311510 | [RKYFG] | 74/1.63 | 0/0.62 | 2/1.06 | 27/0.27 | 33/1.23 | 0 | 0 |
| basic beta-1,3-glucanase [*C. annuum*]/ AAF34761 | [NNA] | 99/1.46 | 0/0.62 | 0/0.64 | 42/0.39 | 27/1.28 | 0 | 8/1.01 |
| chloroplast small heat shock protein class I [*C. frutescens*]/ AAQ19680 |  | 24/1.53 | 0/0.52 | 0/0.41 | 16/0.37 | 1/0.92 | 3/1.15 | 0 |
| enolase [*C. annum*]/ XP_016542903 |  | 182/1.43 | 1/0.73 | 10/0.97 | 37/0.38 | 78/1.70 | 0 | 17/1.23 |
| hypothetical protein BC332_07738 [*Capsicum chinense*]/ PHU22631 |  | 117/1.52 | 1/0.72 | 3/0.79 | 91/0.36 | 10/1.06 | 16/1.15 | 2/0.96 |
| lichenase [C. annum]/ XP_016563240 | [NNA], [PSN] | 107/1.46 | 0/0.62 | 4/0.88 | 33/0.39 | 20/1.28 | 0 | 8/1.01 |
| NADPH-dependent aldehyde reductase 1, chloroplastic [C. annuum]/ XP_047250615 | [NNA] | 59/1.78 | 1/0.85 | 0/0.48 | 44/0.33 | 50/1.52 | 0 | 10/1.26 |
| Oleosin 21.2 kDa [*C. annuum*]/ PHT73078 |  | 37/1.26 | 0/0.54 | 16/1.09 | 47/0.46 | 18/1.46 | 0 | 5/1.19 |
| osmotin-like protein OSML13 precursor [*C. annuum*]/ NP_001311827 |  | 28/1.14 | 0/0.53 | 0/0.34 | 11/0.24 | 3/0.96 | 0 | 2/0.96 |
| pathogenesis-related protein 10 [*C. annuum*]/ AAF63519 | [ENVEG], [NVEG] | 42/1.42 | 0/0.61 | 0/0.44 | 17/0.21 | 17/1.38 | 0 | 7/1.07 |
| pathogenesis-related protein 10 [*C. annuum*]/ CAI51309 |  | 28/1.31 | 0/0.56 | 0/0.51 | 8/0.22 | 0 | 0 | 4/1.08 |
| prunin 1 Pru du 6.0101 [*C. annuum*]/ XP_016570626 | [IAT], [NNA] | 183/1.53 | 10/0.86 | 20/1.39 | 77/0.61 | 12/1.23 | 0 | 2/0.99 |
| putative pathogenesis related protein [*C. chinense*]/ CAI48023 |  | 23/1.72 | 0/0.64 | 1/0.34 | 10/0.21 | 0 | 3/1.03 | 20/1.14 |
| Rubber elongation factor protein [*H. brasiliensis*]/P15252 | epitopes (40 in total):  [AAPEAARSLA], [AEDEDNQQGQ], [AEDEDNQQGQGEGLKYLGF], [ARSLASSLPG], [ASIQVVSAIR], [AVPLYNRFSY], [DIIEGPVKNV], [DKSGPLQPGV], [DNQQGQGEGL], [DRSLPPIVKD], [FSNVYLFAKDKSGPLQPGV], [IDRSLPPIVK], [IPNGALKFVD], [KDASIQVVSA], [KFVDSTVVASVTIIDRSLP], [KYLGFVQDAA], [LKFVDSTVVA], [LQPGVDIIEG], [MAEDEDNQQG], [NRFSYIPNGA], [PGQTKILAKVFY], [PGVDIIEGPVKNV], [PIVKDASIQV], [PVKNVAVPLY], [QGEGLKYLGF], [QPGVDIIEGPVKNVAVPLY], [QTKILAKVFY], [RSLPPIVKDASIQVVSAIR], [SAIRAAPEAARSLASSLPG], [SLPGQTKILAKVFYGEN], [SSLPGQTKIL], [STVVASVTII], [SVTIIDRSLP], [TFSNVYLFAK], [TYAVTTFSNV], [VPLYNRFSYIPNGALKFVD], [VQDAATYAVT], [VSAIRAAPEA], [YLFAKDKSGP], [YLGFVQDAATYAVTTFSNV], [YNRFSYIPNG] | 55/1.73 | 1/0.78 | 0/0.27 | 11/0.18 | 0 | 13/1.16 | 0 |
| serpin-ZX-like [*C. annuum*]/ XP_016568712 |  | 123/1.87 | 0/0.65 | 6/1.04 | 17/0.22 | 31/1.19 | 10/1.24 | 0 |
| stress-related protein [*C. baccatum*]/ PHT38668 |  | 88/1.82 | 0/0.58 | 4/0.78 | 27/0.30 | 12/1.12 | 0 | 10/1.11 |
| suberization-associated anionic peroxidase 2 [*C. annuum*]/ PHT90648 |  | 44/1.32 | 5/0.89 | 0/0.59 | 27/0.38 | 44/1/17 | 0 | 0 |
| vicilin Jug r 2.0101 [*C. annuum*]/ XP_016567997 | [KVVR] | 55/ 1.80 | 7/1.16 | 3/0.87 | 19/0.31 | 19/1.12 | 1/0.92 | 14/1.25 |

^a^Protein name [organism]/NCBI database accession. ^b^IgE epitope on a protein mapped with AlgPred 2.0 web server (https://webs.iiitd.edu.in/raghava/algpred/) - designed for mapping experimentally validated IgE epitopes on the query sequence. ^c^Presence of proinflammatory epitopes in a protein estimated using ProInflam web server (http://metagenomics.iiserb.ac.in/proinflam), with window length 15 and 0.9 threshold: number of epitopes with score ≥ threshold/the best score. ^d^Presence of cytokine inducing peptides estimated for IL-4 (https://webs.iiitd.edu.in/raghava/il4pred/) and IFN-γ (https://webs.iiitd.edu.in/raghava/ifnepitope/) at window length 15 and threshold 0.7, and for IL-6 (https://webs.iiitd.edu.in/raghava/il4pred/) at window length 15 and threshold 0.11: number of epitopes with score ≥ threshold/the best score. ^e^Presence of antibody-specific B-cell epitopes estimated using IgPred web server (https://webs.iiitd.edu.in/raghava/igpred/) at fixed length option which scan a protein to identify IgG-, IgA- or IgE-specific B-cell epitopes.

Supplementary Table 3S. Clinical characteristic of allergic individuals in the study.

| **Individual** | **Ethnicity** | **Age** | **Sex** | **Allergen** | **Ingested food** | **Clinical manifestations** | **Hospitalization** | **Epinephrine** | **Tested** | **Specific IgE^*^** | | | | **Total IgE^**^** |
| --- | --- | --- | --- | --- | --- | --- | --- | --- | --- | --- | --- | --- | --- | --- |
|  |  |  |  |  |  |  |  |  |  | **paprika** | **tomato** | **potato** | **CCD** | **IU/mL** |
|  |  |  |  |  |  |  |  |  |  | **kU/L** | **kU/L** | **kU/L** |  |  |
| 1 | Caucasian | 32 | F | paprika | spicy paprika soup, spice sauce, ketchup | dyspnoea, anaphylaxis | Y | Y | Y | <0.35 | <0.35 | <0.35 | negative | 23 |
| 2 | Caucasian | 44 | F | paprika | pizza, sandwich | oral syndrome | N | N | Y | <0.35 | <0.35 | <0.35 | negative | 228 |
| 3 | Caucasian | 57 | F | paprika | fast food, pizza, ketchup | oral syndrome, rash | N | N | Y | <0.35 | <0.35 | <0.35 | negative | 173 |

*Test: EUROLINE Atopy Screen panel, Allercoat™ 6-ELISA, Allergy profile pollen-food cross reactions testes (EUROIMMUN AG, Lübeck, Germany). Norm for age <0.35 kU/L.

** Test Total IgE (Cat no. EV 3840-9601 E; EUROIMMUN AG, Lübeck, Germany). Norm for age- <100 IU/mL.


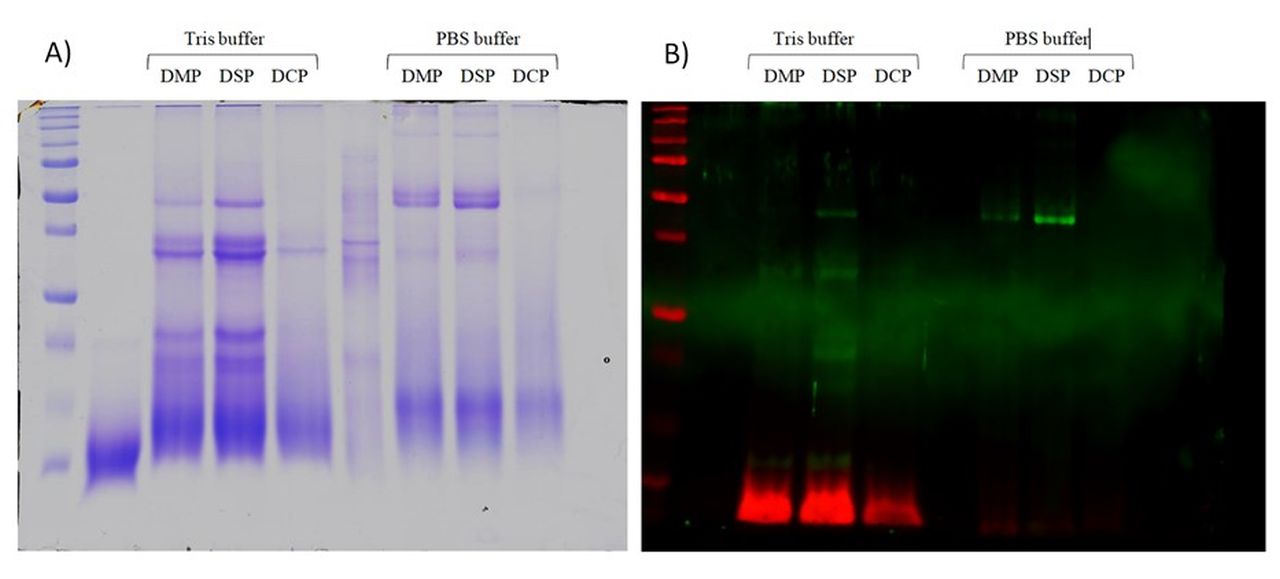


Supplementary Figure S1. Full length SDS-PAGE of protein extracts from paprika spices (A) and their IgE-immunoblotting with human sera (B, green bars). At the bottom of immunoblotting graph, you can see the autofluorescence (red) of the extracts.

*Supplementary data*. All proteomic data obtained by mass spectrometry. Proteins highlighted in blue were analysed and discussed in the manuscript.

| ↑Family | M | DB | Accession | Score | Mass | Matches | Match(sig) | Sequences | Seq(sig) | emPAI | Description |
| --- | --- | --- | --- | --- | --- | --- | --- | --- | --- | --- | --- |
| 1 | 1 | NCBInr | gi\|697173573 | 241 | 96286 | 7 | 7 | 4 | 4 | 0,19 | PREDICTED: vicilin-like antimicrobial peptides 2-3 isoform X1 [Nicotiana tomentosiformis] |
| 1 | 1 | NCBInr | gi\|697173573 | 239 | 96286 | 6 | 6 | 4 | 4 | 0,19 | PREDICTED: vicilin-like antimicrobial peptides 2-3 isoform X1 [Nicotiana tomentosiformis] |
| 2 | 1 | NCBInr | gi\|697173573 | 233 | 96286 | 6 | 6 | 4 | 4 | 0,19 | PREDICTED: vicilin-like antimicrobial peptides 2-3 isoform X1 [Nicotiana tomentosiformis] |
| 13 | 1 | NCBInr | gi\|697173573 | 133 | 96286 | 3 | 3 | 2 | 2 | 0,09 | PREDICTED: vicilin-like antimicrobial peptides 2-3 isoform X1 [Nicotiana tomentosiformis] |
| 9 | 1 | NCBInr | gi\|697173573 | 148 | 96286 | 4 | 4 | 2 | 2 | 0,09 | PREDICTED: vicilin-like antimicrobial peptides 2-3 isoform X1 [Nicotiana tomentosiformis] |
| 1 | 2 | NCBInr | gi\|565390061 | 133 | 66424 | 5 | 5 | 4 | 4 | 0,29 | PREDICTED: vicilin-like antimicrobial peptides 2-1-like [Solanum tuberosum] |
| 1 | 2 | NCBInr | gi\|565390061 | 119 | 66424 | 4 | 4 | 4 | 4 | 0,29 | PREDICTED: vicilin-like antimicrobial peptides 2-1-like [Solanum tuberosum] |
| 2 | 3 | NCBInr | gi\|565390061 | 110 | 66424 | 3 | 3 | 3 | 3 | 0,21 | PREDICTED: vicilin-like antimicrobial peptides 2-1-like [Solanum tuberosum] |
| 13 | 3 | NCBInr | gi\|565390061 | 70 | 66424 | 2 | 2 | 2 | 2 | 0,14 | PREDICTED: vicilin-like antimicrobial peptides 2-1-like [Solanum tuberosum] |
| 9 | 3 | NCBInr | gi\|565390061 | 83 | 66424 | 3 | 3 | 2 | 2 | 0,14 | PREDICTED: vicilin-like antimicrobial peptides 2-1-like [Solanum tuberosum] |
| 3 | 2 | NCBInr | gi\|697189073 | 426 | 59961 | 8 | 8 | 5 | 5 | 0,42 | PREDICTED: 11S globulin subunit beta-like [Nicotiana tomentosiformis] |
| 3 | 3 | NCBInr | gi\|697189073 | 209 | 59961 | 5 | 5 | 4 | 4 | 0,33 | PREDICTED: 11S globulin subunit beta-like [Nicotiana tomentosiformis] |
| 1 | 3 | NCBInr | gi\|697189073 | 162 | 59961 | 4 | 4 | 4 | 4 | 0,33 | PREDICTED: 11S globulin subunit beta-like [Nicotiana tomentosiformis] |
| 3 | 4 | NCBInr | gi\|697189073 | 136 | 59961 | 4 | 4 | 3 | 3 | 0,24 | PREDICTED: 11S globulin subunit beta-like [Nicotiana tomentosiformis] |
| 3 | 4 | NCBInr | gi\|697189073 | 156 | 59961 | 3 | 3 | 2 | 2 | 0,24 | PREDICTED: 11S globulin subunit beta-like [Nicotiana tomentosiformis] |
| 3 | 1 | NCBInr | gi\|460404531 | 468 | 57993 | 7 | 7 | 4 | 4 | 0,34 | PREDICTED: 11S globulin subunit beta [Solanum lycopersicum] |
| 3 | 2 | NCBInr | gi\|460404531 | 215 | 57993 | 4 | 4 | 4 | 4 | 0,34 | PREDICTED: 11S globulin subunit beta [Solanum lycopersicum] |
| 1 | 1 | NCBInr | gi\|460404531 | 241 | 57993 | 5 | 5 | 4 | 4 | 0,34 | PREDICTED: 11S globulin subunit beta [Solanum lycopersicum] |
| 3 | 1 | NCBInr | gi\|460404531 | 356 | 57993 | 6 | 6 | 3 | 3 | 0,24 | PREDICTED: 11S globulin subunit beta [Solanum lycopersicum] |
| 3 | 2 | NCBInr | gi\|460404531 | 175 | 57993 | 3 | 3 | 2 | 2 | 0,24 | PREDICTED: 11S globulin subunit beta [Solanum lycopersicum] |
| 3 | 5 | NCBInr | gi\|460404531 | 109 | 57993 | 2 | 2 | 2 | 2 | 0,16 | PREDICTED: 11S globulin subunit beta [Solanum lycopersicum] |
| 8 | 2 | NCBInr | gi\|565353639 | 110 | 57339 | 2 | 2 | 2 | 2 | 0,16 | PREDICTED: serine carboxypeptidase-like 48-like [Solanum tuberosum] |
| 3 | 3 | NCBInr | gi\|697189071 | 278 | 57152 | 5 | 5 | 3 | 3 | 0,25 | PREDICTED: 11S globulin subunit beta-like [Nicotiana tomentosiformis] |
| 3 | 1 | NCBInr | gi\|697151558 | 218 | 57116 | 5 | 5 | 5 | 5 | 0,45 | PREDICTED: legumin B-like [Nicotiana tomentosiformis] |
| 1 | 2 | NCBInr | gi\|697151558 | 186 | 57116 | 5 | 5 | 5 | 5 | 0,45 | PREDICTED: legumin B-like [Nicotiana tomentosiformis] |
| 3 | 2 | NCBInr | gi\|697151558 | 195 | 57116 | 6 | 6 | 4 | 4 | 0,34 | PREDICTED: legumin B-like [Nicotiana tomentosiformis] |
| 3 | 1 | NCBInr | gi\|697151558 | 199 | 57116 | 4 | 4 | 3 | 3 | 0,34 | PREDICTED: legumin B-like [Nicotiana tomentosiformis] |
| 3 | 2 | NCBInr | gi\|697151558 | 292 | 57116 | 5 | 5 | 3 | 3 | 0,25 | PREDICTED: legumin B-like [Nicotiana tomentosiformis] |
| 3 | 5 | NCBInr | gi\|698538021 | 124 | 56908 | 4 | 4 | 4 | 4 | 0,35 | PREDICTED: legumin B-like [Nicotiana sylvestris] |
| 8 | 1 | NCBInr | gi\|460411135 | 129 | 56908 | 2 | 2 | 2 | 2 | 0,16 | PREDICTED: serine carboxypeptidase-like [Solanum lycopersicum] |
| 8 | 3 | NCBInr | gi\|148469861 | 86 | 56793 | 3 | 3 | 2 | 2 | 0,25 | serine carboxypeptidase III [Nicotiana tabacum] |
| 10 | 1 | NCBInr | gi\|148469861 | 161 | 56793 | 2 | 2 | 2 | 2 | 0,16 | serine carboxypeptidase III [Nicotiana tabacum] |
| 3 | 3 | NCBInr | gi\|460376510 | 274 | 56393 | 6 | 6 | 4 | 4 | 0,35 | PREDICTED: legumin B-like [Solanum lycopersicum] |
| 3 | 3 | NCBInr | gi\|460376510 | 167 | 56393 | 4 | 4 | 3 | 3 | 0,35 | PREDICTED: legumin B-like [Solanum lycopersicum] |
| 3 | 3 | NCBInr | gi\|460376510 | 143 | 56393 | 3 | 3 | 2 | 2 | 0,16 | PREDICTED: legumin B-like [Solanum lycopersicum] |
| 4 | 1 | NCBInr | gi\|697139889 | 179 | 55964 | 4 | 4 | 3 | 3 | 0,25 | PREDICTED: 11S globulin seed storage protein 2-like [Nicotiana tomentosiformis] |
| 2 | 2 | NCBInr | gi\|697139889 | 254 | 55964 | 4 | 4 | 3 | 3 | 0,25 | PREDICTED: 11S globulin seed storage protein 2-like [Nicotiana tomentosiformis] |
| 3 | 1 | NCBInr | gi\|697139889 | 202 | 55964 | 4 | 4 | 3 | 3 | 0,26 | PREDICTED: 11S globulin seed storage protein 2-like [Nicotiana tomentosiformis] |
| 2 | 1 | NCBInr | gi\|697139889 | 225 | 55964 | 3 | 3 | 2 | 2 | 0,16 | PREDICTED: 11S globulin seed storage protein 2-like [Nicotiana tomentosiformis] |
| 2 | 1 | NCBInr | gi\|698469161 | 510 | 55749 | 10 | 10 | 5 | 5 | 0,7 | PREDICTED: 11S globulin seed storage protein 2-like [Nicotiana sylvestris] |
| 19 | 2 | NCBInr | gi\|698469161 | 102 | 55749 | 3 | 3 | 3 | 3 | 0,25 | PREDICTED: 11S globulin seed storage protein 2-like [Nicotiana sylvestris] |
| 3 | 4 | NCBInr | gi\|565379404 | 142 | 55173 | 6 | 6 | 5 | 5 | 0,47 | PREDICTED: legumin B-like [Solanum tuberosum] |
| 1 | 4 | NCBInr | gi\|565379404 | 132 | 55173 | 5 | 5 | 5 | 5 | 0,47 | PREDICTED: legumin B-like [Solanum tuberosum] |
| 3 | 4 | NCBInr | gi\|565379404 | 169 | 55173 | 5 | 5 | 4 | 4 | 0,36 | PREDICTED: legumin B-like [Solanum tuberosum] |
| 3 | 4 | NCBInr | gi\|565379404 | 158 | 55173 | 5 | 5 | 4 | 4 | 0,36 | PREDICTED: legumin B-like [Solanum tuberosum] |
| 3 | 1 | NCBInr | gi\|565379404 | 233 | 55173 | 7 | 7 | 4 | 4 | 0,36 | PREDICTED: legumin B-like [Solanum tuberosum] |
| 42 | 1 | NCBInr | gi\|840729 | 71 | 54948 | 2 | 2 | 2 | 2 | 0,17 | putative chromoplastic oxydo-reductase [Capsicum annuum] |
| 2 | 2 | NCBInr | gi\|460404101 | 376 | 54717 | 8 | 8 | 4 | 4 | 0,47 | PREDICTED: 11S globulin seed storage protein 2-like [Solanum lycopersicum] |
| 2 | 3 | NCBInr | gi\|460404101 | 196 | 54717 | 5 | 5 | 4 | 4 | 0,36 | PREDICTED: 11S globulin seed storage protein 2-like [Solanum lycopersicum] |
| 4 | 2 | NCBInr | gi\|460404101 | 122 | 54717 | 3 | 3 | 3 | 3 | 0,26 | PREDICTED: 11S globulin seed storage protein 2-like [Solanum lycopersicum] |
| 2 | 4 | NCBInr | gi\|565370161 | 287 | 54678 | 6 | 6 | 3 | 3 | 0,36 | PREDICTED: 11S globulin seed storage protein 2-like [Solanum tuberosum] |
| 2 | 1 | NCBInr | gi\|565370157 | 274 | 54057 | 5 | 5 | 4 | 4 | 0,37 | PREDICTED: 11S globulin seed storage protein 2-like [Solanum tuberosum] |
| 19 | 1 | NCBInr | gi\|565370157 | 104 | 54057 | 2 | 2 | 2 | 2 | 0,17 | PREDICTED: 11S globulin seed storage protein 2-like [Solanum tuberosum] |
| 2 | 2 | NCBInr | gi\|565370157 | 221 | 54057 | 3 | 3 | 2 | 2 | 0,17 | PREDICTED: 11S globulin seed storage protein 2-like [Solanum tuberosum] |
| 2 | 3 | NCBInr | gi\|723733549 | 335 | 54022 | 15 | 15 | 5 | 5 | 0,6 | PREDICTED: LOW QUALITY PROTEIN: 11S globulin seed storage protein 2-like [Solanum lycopersicum] |
| 1 | 2 | NCBInr | gi\|697139896 | 377 | 53916 | 7 | 7 | 4 | 4 | 0,48 | PREDICTED: legumin A-like [Nicotiana tomentosiformis] |
| 1 | 2 | NCBInr | gi\|697139891 | 235 | 53745 | 8 | 8 | 3 | 3 | 0,27 | PREDICTED: legumin A-like [Nicotiana tomentosiformis] |
| 10 | 2 | NCBInr | gi\|697139891 | 94 | 53745 | 3 | 3 | 2 | 2 | 0,17 | PREDICTED: legumin A-like [Nicotiana tomentosiformis] |
| 1 | 2 | NCBInr | gi\|697139891 | 128 | 53745 | 4 | 4 | 2 | 2 | 0,17 | PREDICTED: legumin A-like [Nicotiana tomentosiformis] |
| 1 | 1 | NCBInr | gi\|460402890 | 643 | 53649 | 32 | 32 | 7 | 7 | 0,74 | PREDICTED: 12S seed storage protein CRA1-like [Solanum lycopersicum] |
| 1 | 1 | NCBInr | gi\|460402890 | 297 | 53649 | 12 | 12 | 6 | 6 | 0,61 | PREDICTED: 12S seed storage protein CRA1-like [Solanum lycopersicum] |
| 1 | 1 | NCBInr | gi\|460402890 | 524 | 53649 | 24 | 23 | 5 | 5 | 0,48 | PREDICTED: 12S seed storage protein CRA1-like [Solanum lycopersicum] |
| 4 | 1 | NCBInr | gi\|460402890 | 200 | 53649 | 7 | 7 | 5 | 5 | 0,48 | PREDICTED: 12S seed storage protein CRA1-like [Solanum lycopersicum] |
| 10 | 1 | NCBInr | gi\|460402890 | 130 | 53649 | 5 | 4 | 4 | 3 | 0,27 | PREDICTED: 12S seed storage protein CRA1-like [Solanum lycopersicum] |
| 4 | 1 | NCBInr | gi\|460402890 | 182 | 53649 | 5 | 5 | 3 | 3 | 0,27 | PREDICTED: 12S seed storage protein CRA1-like [Solanum lycopersicum] |
| 1 | 3 | NCBInr | gi\|697094346 | 114 | 50705 | 4 | 4 | 3 | 3 | 0,28 | PREDICTED: vicilin-like antimicrobial peptides 2-1 [Nicotiana tomentosiformis] |
| 1 | 3 | NCBInr | gi\|697094346 | 107 | 50705 | 3 | 3 | 3 | 3 | 0,28 | PREDICTED: vicilin-like antimicrobial peptides 2-1 [Nicotiana tomentosiformis] |
| 2 | 2 | NCBInr | gi\|697094346 | 112 | 50705 | 3 | 3 | 3 | 3 | 0,28 | PREDICTED: vicilin-like antimicrobial peptides 2-1 [Nicotiana tomentosiformis] |
| 13 | 2 | NCBInr | gi\|697094346 | 73 | 50705 | 2 | 2 | 2 | 2 | 0,18 | PREDICTED: vicilin-like antimicrobial peptides 2-1 [Nicotiana tomentosiformis] |
| 9 | 2 | NCBInr | gi\|697094346 | 86 | 50705 | 3 | 3 | 2 | 2 | 0,18 | PREDICTED: vicilin-like antimicrobial peptides 2-1 [Nicotiana tomentosiformis] |
| 7 | 2 | NCBInr | gi\|3328122 | 118 | 50594 | 2 | 2 | 2 | 2 | 0,18 | phosphoglycerate kinase precursor [Solanum tuberosum] |
| 16 | 1 | NCBInr | gi\|460368352 | 86 | 48487 | 2 | 2 | 2 | 2 | 0,19 | PREDICTED: protein ASPARTIC PROTEASE IN GUARD CELL 2-like [Solanum lycopersicum] |
| 22 | 1 | NCBInr | gi\|460368352 | 95 | 48487 | 2 | 2 | 2 | 2 | 0,19 | PREDICTED: protein ASPARTIC PROTEASE IN GUARD CELL 2-like [Solanum lycopersicum] |
| 2 | 1 | NCBInr | gi\|2078350 | 211 | 48178 | 4 | 4 | 4 | 4 | 0,42 | transaldolase [Solanum tuberosum] |
| 2 | 2 | NCBInr | gi\|217795376 | 190 | 48110 | 4 | 4 | 4 | 4 | 0,42 | transaldolase [Dimocarpus longan] |
| 12 | 1 | NCBInr | gi\|350538295 | 117 | 48054 | 2 | 2 | 2 | 2 | 0,19 | enolase [Solanum lycopersicum] |
| 6 | 1 | NCBInr | gi\|565376892 | 163 | 42756 | 4 | 4 | 4 | 4 | 0,48 | PREDICTED: serpin-ZX-like [Solanum tuberosum] |
| 7 | 1 | NCBInr | gi\|3738257 | 156 | 42517 | 2 | 2 | 2 | 2 | 0,22 | cytosolic phosphoglycerate kinase 1 [Populus nigra] |
| 38 | 1 | NCBInr | gi\|460393933 | 58 | 41939 | 2 | 2 | 2 | 2 | 0,22 | PREDICTED: chitotriosidase-1 [Solanum lycopersicum] |
| 5 | 2 | NCBInr | gi\|115461639 | 140 | 41897 | 5 | 5 | 5 | 5 | 0,65 | Os05g0106600 [Oryza sativa Japonica Group] |
| 11 | 1 | NCBInr | gi\|565394146 | 157 | 39549 | 3 | 3 | 3 | 3 | 0,38 | PREDICTED: GDSL esterase/lipase At1g71250-like [Solanum tuberosum] |
| 46 | 1 | NCBInr | gi\|6984122 | 68 | 39487 | 1 | 1 | 1 | 1 | 0,11 | basic beta-1,3-glucanase [Capsicum annuum] |
| 4 | 1 | NCBInr | gi\|460373941 | 232 | 39093 | 6 | 6 | 5 | 5 | 0,72 | PREDICTED: suberization-associated anionic peroxidase 2 [Solanum lycopersicum] |
| 5 | 1 | NCBInr | gi\|3219762 | 166 | 37521 | 5 | 5 | 5 | 5 | 0,75 | RecName: Full=Actin-54, partial [Nicotiana tabacum] |
| 12 | 1 | NCBInr | gi\|460368658 | 155 | 37492 | 3 | 3 | 3 | 3 | 0,4 | PREDICTED: glucose and ribitol dehydrogenase homolog 1 [Solanum lycopersicum] |
| 9 | 1 | NCBInr | gi\|1071660 | 169 | 35943 | 4 | 4 | 4 | 4 | 0,6 | annexin [Capsicum annuum] |
| 4 | 1 | NCBInr | gi\|460761 | 332 | 35295 | 9 | 9 | 8 | 8 | 1,59 | fibrillin [Capsicum annuum] |
| 5 | 1 | NCBInr | gi\|460761 | 175 | 35295 | 4 | 4 | 4 | 4 | 0,61 | fibrillin [Capsicum annuum] |
| 7 | 1 | NCBInr | gi\|460761 | 167 | 35295 | 3 | 3 | 3 | 3 | 0,43 | fibrillin [Capsicum annuum] |
| 25 | 1 | NCBInr | gi\|460761 | 67 | 35295 | 1 | 1 | 1 | 1 | 0,13 | fibrillin [Capsicum annuum] |
| 11 | 1 | NCBInr | gi\|460368710 | 125 | 35079 | 2 | 2 | 2 | 2 | 0,27 | PREDICTED: desiccation-related protein PCC13-62-like [Solanum lycopersicum] |
| 5 | 1 | NCBInr | gi\|460368710 | 249 | 35079 | 3 | 3 | 2 | 2 | 0,27 | PREDICTED: desiccation-related protein PCC13-62-like [Solanum lycopersicum] |
| 16 | 1 | NCBInr | gi\|565364618 | 123 | 34153 | 3 | 3 | 3 | 3 | 0,45 | PREDICTED: probable xyloglucan endotransglucosylase/hydrolase protein 32-like [Solanum tuberosum] |
| 11 | 1 | NCBInr | gi\|565364618 | 108 | 34153 | 3 | 3 | 3 | 3 | 0,45 | PREDICTED: probable xyloglucan endotransglucosylase/hydrolase protein 32-like [Solanum tuberosum] |
| 17 | 1 | NCBInr | gi\|565364618 | 104 | 34153 | 2 | 2 | 2 | 2 | 0,28 | PREDICTED: probable xyloglucan endotransglucosylase/hydrolase protein 32-like [Solanum tuberosum] |
| 16 | 2 | NCBInr | gi\|697130904 | 87 | 33829 | 3 | 3 | 3 | 3 | 0,45 | PREDICTED: probable xyloglucan endotransglucosylase/hydrolase protein 31 [Nicotiana tomentosiformis] |
| 6 | 1 | NCBInr | gi\|58578270 | 160 | 31471 | 4 | 4 | 4 | 4 | 0,7 | anionic peroxidase [Capsicum chinense] |
| 28 | 1 | NCBInr | gi\|5739347 | 88 | 30487 | 2 | 2 | 2 | 2 | 0,32 | hypersensitive response assisting protein [Capsicum annuum] |
| 32 | 1 | NCBInr | gi\|5739347 | 61 | 30487 | 1 | 1 | 1 | 1 | 0,15 | hypersensitive response assisting protein [Capsicum annuum] |
| 9 | 1 | NCBInr | gi\|170676242 | 131 | 30013 | 4 | 4 | 4 | 4 | 0,75 | PGIP [Capsicum annuum] |
| 7 | 1 | NCBInr | gi\|170676242 | 202 | 30013 | 5 | 5 | 4 | 4 | 0,75 | PGIP [Capsicum annuum] |
| 15 | 1 | NCBInr | gi\|170676242 | 82 | 30013 | 2 | 2 | 2 | 2 | 0,32 | PGIP [Capsicum annuum] |
| 69 | 1 | NCBInr | gi\|170676242 | 53 | 30013 | 1 | 1 | 1 | 1 | 0,15 | PGIP [Capsicum annuum] |
| 23 | 1 | NCBInr | gi\|407907615 | 95 | 29972 | 3 | 3 | 3 | 3 | 0,52 | thioredoxin peroxidase, partial [Nicotiana tabacum] |
| 23 | 2 | NCBInr | gi\|460407951 | 73 | 29728 | 3 | 3 | 3 | 3 | 0,53 | PREDICTED: 2-Cys peroxiredoxin BAS1, chloroplastic [Solanum lycopersicum] |
| 14 | 1 | NCBInr | gi\|6048277 | 130 | 29449 | 3 | 3 | 3 | 3 | 0,53 | pectin methylesterase [Nicotiana tabacum] |
| 8 | 1 | NCBInr | gi\|697185054 | 175 | 27597 | 4 | 4 | 3 | 3 | 0,57 | PREDICTED: late embryogenesis abundant protein D-29-like [Nicotiana tomentosiformis] |
| 8 | 1 | NCBInr | gi\|697185054 | 156 | 27597 | 3 | 3 | 3 | 3 | 0,58 | PREDICTED: late embryogenesis abundant protein D-29-like [Nicotiana tomentosiformis] |
| 31 | 1 | NCBInr | gi\|15419836 | 85 | 27254 | 1 | 1 | 1 | 1 | 0,17 | thaumatin-like protein [Capsicum annuum] |
| 15 | 1 | NCBInr | gi\|460404385 | 127 | 26867 | 2 | 2 | 2 | 2 | 0,37 | PREDICTED: late embryogenesis abundant protein D-34-like [Solanum lycopersicum] |
| 65 | 1 | NCBInr | gi\|37904866 | 54 | 26237 | 1 | 1 | 1 | 1 | 0,17 | small heat shock protein [Capsicum annuum] |
| 21 | 2 | NCBInr | gi\|675356205 | 89 | 25706 | 2 | 2 | 2 | 2 | 0,39 | Histone H3.1 [Auxenochlorella protothecoides] |
| 18 | 1 | NCBInr | gi\|15235889 | 103 | 25193 | 2 | 2 | 2 | 2 | 0,4 | 20S proteasome subunit PBA1 [Arabidopsis thaliana] |
| 92 | 1 | NCBInr | gi\|24061762 | 46 | 25016 | 1 | 1 | 1 | 1 | 0,18 | glutathione S-transferase [Capsicum annuum] |
| 13 | 1 | NCBInr | gi\|299891471 | 122 | 24099 | 2 | 2 | 2 | 2 | 0,42 | mitochondrial small heat shock protein [Capsicum annuum] |
| 18 | 1 | NCBInr | gi\|299891471 | 81 | 24099 | 1 | 1 | 1 | 1 | 0,19 | mitochondrial small heat shock protein [Capsicum annuum] |
| 20 | 1 | NCBInr | gi\|460380798 | 100 | 21524 | 2 | 2 | 2 | 2 | 0,48 | PREDICTED: 22.7 kDa class IV heat shock protein-like [Solanum lycopersicum] |
| 64 | 1 | NCBInr | gi\|50236424 | 54 | 21414 | 1 | 1 | 1 | 1 | 0,22 | lipocalin protein [Capsicum annuum] |
| 11 | 1 | NCBInr | gi\|460412845 | 124 | 18183 | 3 | 3 | 3 | 3 | 0,99 | PREDICTED: oleosin 5-like [Solanum lycopersicum] |
| 4 | 1 | NCBInr | gi\|590589403 | 190 | 18024 | 6 | 6 | 5 | 5 | 2,19 | HSP20-like chaperones superfamily protein [Theobroma cacao] |
| 4 | 3 | NCBInr | gi\|565390171 | 78 | 17523 | 4 | 4 | 4 | 4 | 1,59 | PREDICTED: 17.4 kDa class I heat shock protein-like [Solanum tuberosum] |
| 6 | 1 | NCBInr | gi\|42564093 | 222 | 17521 | 3 | 3 | 2 | 2 | 0,61 | basic beta-1,3-glucanase [Capsicum annuum] |
| 10 | 1 | NCBInr | gi\|18654477 | 127 | 17509 | 3 | 3 | 3 | 3 | 1,04 | thioredoxin peroxidase [Capsicum annuum] |
| 4 | 2 | NCBInr | gi\|658016939 | 165 | 17485 | 5 | 5 | 4 | 4 | 1,59 | PREDICTED: 17.8 kDa class I heat shock protein-like [Malus domestica] |
| 34 | 1 | NCBInr | gi\|225429598 | 80 | 17441 | 2 | 2 | 2 | 2 | 0,61 | PREDICTED: 17.3 kDa class II heat shock protein [Vitis vinifera] |
| 29 | 1 | NCBInr | gi\|60542787 | 85 | 17411 | 1 | 1 | 1 | 1 | 0,27 | pathogenesis-related protein 10 [Capsicum chinense] |
| 44 | 1 | NCBInr | gi\|7542607 | 70 | 17390 | 1 | 1 | 1 | 1 | 0,27 | pathogenesis-related protein 10 [Capsicum annuum] |
| 9 | 1 | NCBInr | gi\|75279028 | 142 | 17311 | 3 | 2 | 3 | 2 | 0,62 | RecName: Full=17.3 kDa class II heat shock protein; AltName: Full=Hsp17.3; AltName: Full=Hsp20.2 [Solanum peruvianum] |
| 15 | 1 | NCBInr | gi\|58531054 | 105 | 17274 | 3 | 3 | 3 | 3 | 1,06 | putative pathogenesis related protein [Capsicum chinense] |
| 17 | 1 | NCBInr | gi\|122022 | 114 | 16423 | 2 | 2 | 2 | 2 | 0,66 | RecName: Full=Histone H2B.1 [Triticum aestivum] |
| 21 | 1 | NCBInr | gi\|121982 | 99 | 16060 | 2 | 2 | 2 | 2 | 0,68 | RecName: Full=Histone H2A.2.2 [Triticum aestivum] |
| 8 | 1 | NCBInr | gi\|1617013 | 161 | 15724 | 4 | 4 | 3 | 3 | 1,21 | histone H2B like protein [Arabidopsis thaliana] |
| 10 | 1 | NCBInr | gi\|132270 | 116 | 14713 | 3 | 3 | 3 | 3 | 1,32 | RecName: Full=Rubber elongation factor protein; Short=REF; AltName: Allergen=Hev b 1 [Hevea brasiliensis] |
| 5 | 1 | NCBInr | gi\|132270 | 138 | 14713 | 3 | 3 | 3 | 3 | 1,33 | RecName: Full=Rubber elongation factor protein; Short=REF; AltName: Allergen=Hev b 1 [Hevea brasiliensis] |
| 29 | 1 | NCBInr | gi\|40794497 | 76 | 13175 | 1 | 1 | 1 | 1 | 0,37 | chitinase class I [Capsicum annuum] |
| 12 | 1 | NCBInr | gi\|460367790 | 123 | 12303 | 2 | 2 | 2 | 2 | 0,95 | PREDICTED: non-specific lipid-transfer protein A-like [Solanum lycopersicum] |
| 14 | 1 | NCBInr | gi\|224293 | 122 | 11329 | 2 | 2 | 2 | 2 | 1,07 | histone H4 |
| 18 | 1 | NCBInr | gi\|159464880 | 107 | 11062 | 3 | 3 | 2 | 2 | 1,09 | histone H4 [Chlamydomonas reinhardtii] |
| 5 | 1 | NCBInr | gi\|698431723 | 189 | 10997 | 4 | 4 | 3 | 3 | 2,07 | PREDICTED: em protein H5 [Nicotiana sylvestris] |
| 7 | 1 | NCBInr | gi\|460390471 | 158 | 10030 | 3 | 3 | 3 | 3 | 2,42 | PREDICTED: em protein H5 [Solanum lycopersicum] |
| 5 | 2 | NCBInr | gi\|460390471 | 136 | 10030 | 2 | 2 | 2 | 2 | 1,27 | PREDICTED: em protein H5 [Solanum lycopersicum] |
| 4 | 4 | NCBInr | gi\|163311856 | 77 | 9291 | 2 | 2 | 2 | 2 | 1,4 | small heat shock protein class I [Capsicum annuum] |
| 8 | 2 | NCBInr | gi\|114384147 | 103 | 8133 | 2 | 2 | 2 | 2 | 1,72 | LEA protein 4 [Capsicum annuum] |
| 25 | 1 | NCBInr | gi\|1644306 | 87 | 6754 | 2 | 2 | 2 | 2 | 2,27 | viral envelope protein [Nicotiana tabacum] |
| 16 | 1 | NCBInr | gi\|1644306 | 105 | 6754 | 2 | 2 | 2 | 2 | 2,28 | viral envelope protein [Nicotiana tabacum] |
| 17 | 1 | NCBInr | gi\|1644306 | 77 | 6754 | 2 | 2 | 2 | 2 | 2,27 | viral envelope protein [Nicotiana tabacum] |
| 13 | 1 | NCBInr | gi\|18499 | 103 | 11965 | 1 | 1 | 1 | 1 | 0,41 | late embryogenesis abundant protein [Gossypium hirsutum] |
| 14 | 1 | NCBInr | gi\|729405394 | 98 | 53184 | 1 | 1 | 1 | 1 | 0,08 | PREDICTED: cruciferin PGCRURSE5 [Tarenaya hassleriana] |
| 15 | 1 | NCBInr | gi\|120666 | 94 | 36776 | 1 | 1 | 1 | 1 | 0,12 | RecName: Full=Glyceraldehyde-3-phosphate dehydrogenase, cytosolic [Antirrhinum majus] |
| 17 | 1 | NCBInr | gi\|697103067 | 81 | 36534 | 1 | 1 | 1 | 1 | 0,12 | PREDICTED: peroxidase 3-like [Nicotiana tomentosiformis] |
| 19 | 1 | NCBInr | gi\|303844 | 79 | 47187 | 1 | 1 | 1 | 1 | 0,09 | eukaryotic initiation factor 4A [Oryza sativa Japonica Group] |
| 20 | 1 | NCBInr | gi\|30526289 | 79 | 29454 | 1 | 1 | 1 | 1 | 0,15 | 29 kDa chitinase-like thermal hysteresis protein [Solanum dulcamara] |
| 21 | 1 | NCBInr | gi\|170243 | 78 | 40572 | 1 | 1 | 1 | 1 | 0,11 | beta (1,3)-glucanase regulator [Nicotiana plumbaginifolia] |
| 22 | 1 | NCBInr | gi\|21481 | 72 | 9285 | 1 | 1 | 1 | 1 | 0,55 | 70-kD heat shock protein [Solanum tuberosum] |
| 23 | 1 | NCBInr | gi\|159464880 | 71 | 11062 | 2 | 2 | 1 | 1 | 0,45 | histone H4 [Chlamydomonas reinhardtii] |
| 24 | 1 | NCBInr | gi\|527205827 | 67 | 76721 | 1 | 1 | 1 | 1 | 0,06 | hypothetical protein M569_03185 [Genlisea aurea] |
| 26 | 1 | NCBInr | gi\|674244918 | 66 | 571656 | 1 | 1 | 1 | 1 | 0,01 | hypothetical protein AALP_AA3G015100 [Arabis alpina] |
| 27 | 1 | NCBInr | gi\|15239136 | 65 | 13272 | 1 | 1 | 1 | 1 | 0,36 | thioredoxin H3 [Arabidopsis thaliana] |
| 28 | 1 | NCBInr | gi\|566161591 | 64 | 26646 | 1 | 1 | 1 | 1 | 0,17 | hypothetical protein POPTR_0003s08470g [Populus trichocarpa] |
| 29 | 1 | NCBInr | gi\|4510395 | 64 | 92890 | 1 | 1 | 1 | 1 | 0,05 | putative beta-galactosidase [Arabidopsis thaliana] |
| 30 | 1 | NCBInr | gi\|302771612 | 63 | 89174 | 1 | 1 | 1 | 1 | 0,05 | hypothetical protein SELMODRAFT_410159 [Selaginella moellendorffii] |
| 31 | 1 | NCBInr | gi\|12322392 | 62 | 165510 | 1 | 1 | 1 | 1 | 0,03 | unknown protein; 42586-33527 [Arabidopsis thaliana] |
| 33 | 1 | NCBInr | gi\|449513032 | 61 | 57669 | 1 | 1 | 1 | 1 | 0,08 | PREDICTED: serine carboxypeptidase-like 49-like [Cucumis sativus] |
| 34 | 1 | NCBInr | gi\|586673518 | 60 | 12763 | 1 | 1 | 1 | 1 | 0,38 | hypothetical protein AMTR_s00100p00159440, partial [Amborella trichopoda] |
| 35 | 1 | NCBInr | gi\|1644306 | 60 | 6754 | 1 | 1 | 1 | 1 | 0,81 | viral envelope protein [Nicotiana tabacum] |
| 36 | 1 | NCBInr | gi\|604312292 | 59 | 49277 | 1 | 1 | 1 | 1 | 0,09 | hypothetical protein MIMGU_mgv1a024803mg, partial [Erythranthe guttata] |
| 37 | 1 | NCBInr | gi\|719968548 | 59 | 22760 | 1 | 1 | 1 | 1 | 0,2 | PREDICTED: vicilin-like antimicrobial peptides 2-2 [Nelumbo nucifera] |
| 39 | 1 | NCBInr | gi\|460367790 | 56 | 12303 | 1 | 1 | 1 | 1 | 0,4 | PREDICTED: non-specific lipid-transfer protein A-like [Solanum lycopersicum] |
| 40 | 1 | NCBInr | gi\|1616628 | 56 | 20764 | 1 | 1 | 1 | 1 | 0,22 | sts15 [Solanum tuberosum] |
| 41 | 1 | NCBInr | gi\|657985907 | 55 | 133673 | 1 | 1 | 1 | 1 | 0,03 | PREDICTED: putative disease resistance protein At3g14460 [Malus domestica] |
| 42 | 1 | NCBInr | gi\|159463262 | 53 | 118165 | 1 | 1 | 1 | 1 | 0,04 | predicted protein [Chlamydomonas reinhardtii] |
| 43 | 1 | NCBInr | gi\|255082530 | 53 | 111061 | 1 | 1 | 1 | 1 | 0,04 | predicted protein [Micromonas sp. RCC299] |
| 44 | 1 | NCBInr | gi\|674895785 | 52 | 30918 | 2 | 2 | 1 | 1 | 0,15 | BnaA04g26030D [Brassica napus] |
| 45 | 1 | NCBInr | gi\|470124256 | 51 | 147681 | 1 | 1 | 1 | 1 | 0,03 | PREDICTED: condensin-2 complex subunit D3-like [Fragaria vesca subsp. vesca] |
| 46 | 1 | NCBInr | gi\|108710478 | 51 | 88602 | 1 | 1 | 1 | 1 | 0,05 | LOC495012 protein, putative, expressed [Oryza sativa Japonica Group] |
| 47 | 1 | NCBInr | gi\|545370097 | 51 | 93458 | 1 | 1 | 1 | 1 | 0,05 | hypothetical protein COCSUDRAFT_65136 [Coccomyxa subellipsoidea C-169] |
| 48 | 1 | NCBInr | gi\|1263291 | 49 | 41748 | 1 | 1 | 1 | 1 | 0,11 | alcohol dehydrogenase 2b [Gossypium hirsutum] |
| 49 | 1 | NCBInr | gi\|674873875 | 48 | 96410 | 1 | 1 | 1 | 1 | 0,04 | BnaC07g51120D [Brassica napus] |
| 50 | 1 | NCBInr | gi\|126077 | 48 | 33472 | 1 | 1 | 1 | 1 | 0,13 | RecName: Full=Late embryogenesis abundant protein D-29; Short=LEA D-29 [Gossypium hirsutum] |
| 51 | 1 | NCBInr | gi\|303271575 | 48 | 136008 | 1 | 1 | 1 | 1 | 0,03 | predicted protein [Micromonas pusilla CCMP1545] |
| 52 | 1 | NCBInr | gi\|15239085 | 48 | 84825 | 1 | 1 | 1 | 1 | 0,05 | pentatricopeptide repeat-containing protein [Arabidopsis thaliana] |
| 53 | 1 | NCBInr | gi\|527191418 | 48 | 53784 | 1 | 1 | 1 | 1 | 0,08 | hypothetical protein M569_12535, partial [Genlisea aurea] |
| 54 | 1 | NCBInr | gi\|2827711 | 48 | 110899 | 1 | 1 | 1 | 1 | 0,04 | oxoglutarate dehydrogenase - like protein [Arabidopsis thaliana] |
| 55 | 1 | NCBInr | gi\|294461233 | 47 | 28036 | 1 | 1 | 1 | 1 | 0,16 | unknown [Picea sitchensis] |
| 56 | 1 | NCBInr | gi\|674938212 | 47 | 33060 | 1 | 1 | 1 | 1 | 0,14 | BnaC09g16300D [Brassica napus] |
| 57 | 1 | NCBInr | gi\|527202013 | 47 | 242889 | 1 | 1 | 1 | 1 | 0,02 | hypothetical protein M569_05834 [Genlisea aurea] |
| 58 | 1 | NCBInr | gi\|147766466 | 47 | 30370 | 1 | 1 | 1 | 1 | 0,15 | hypothetical protein VITISV_034331 [Vitis vinifera] |
| 59 | 1 | NCBInr | gi\|514783407 | 46 | 62278 | 1 | 1 | 1 | 1 | 0,07 | PREDICTED: type I inositol 1,4,5-trisphosphate 5-phosphatase CVP2-like isoform X3 [Setaria italica] |
| 60 | 1 | NCBInr | gi\|629113167 | 46 | 126348 | 1 | 1 | 1 | 1 | 0,03 | hypothetical protein EUGRSUZ_D02334 [Eucalyptus grandis] |
| 61 | 1 | NCBInr | gi\|15226197 | 46 | 79284 | 1 | 1 | 1 | 1 | 0,05 | leucine-rich repeat transmembrane protein kinase-like protein [Arabidopsis thaliana] |
| 62 | 1 | NCBInr | gi\|565366241 | 45 | 55441 | 1 | 1 | 1 | 1 | 0,08 | PREDICTED: uncharacterized protein LOC102600818 [Solanum tuberosum] |
| 63 | 1 | NCBInr | gi\|672189919 | 44 | 65337 | 1 | 1 | 1 | 1 | 0,07 | PREDICTED: globulin-1 S allele [Phoenix dactylifera] |
| 64 | 1 | NCBInr | gi\|302788983 | 44 | 34013 | 1 | 1 | 1 | 1 | 0,13 | hypothetical protein SELMODRAFT_443146 [Selaginella moellendorffii] |
| 65 | 1 | NCBInr | gi\|227438221 | 44 | 104329 | 1 | 1 | 1 | 1 | 0,04 | disease resistance protein [Brassica rapa subsp. pekinensis] |
| 66 | 1 | NCBInr | gi\|565450543 | 44 | 42944 | 1 | 1 | 1 | 1 | 0,1 | hypothetical protein CARUB_v10007243mg, partial [Capsella rubella] |
| 67 | 1 | NCBInr | gi\|727529875 | 44 | 29312 | 1 | 1 | 1 | 1 | 0,15 | PREDICTED: ethylene-responsive transcription factor 1A-like [Camelina sativa] |
| 68 | 1 | NCBInr | gi\|167997063 | 44 | 26626 | 1 | 1 | 1 | 1 | 0,17 | predicted protein [Physcomitrella patens] |
| 69 | 1 | NCBInr | gi\|121982 | 43 | 16060 | 1 | 1 | 1 | 1 | 0,29 | RecName: Full=Histone H2A.2.2 [Triticum aestivum] |
| 70 | 1 | NCBInr | gi\|308803310 | 43 | 27367 | 1 | 1 | 1 | 1 | 0,17 | unnamed protein product [Ostreococcus tauri] |
| 71 | 1 | NCBInr | gi\|672127489 | 43 | 157518 | 1 | 1 | 1 | 1 | 0,03 | PREDICTED: formin-like protein 5 [Phoenix dactylifera] |
| 2 | 1 | NCBInr | gi\|302771612 | 476 | 89174 | 29 | 29 | 1 | 1 | 0,1 | hypothetical protein SELMODRAFT_410159 [Selaginella moellendorffii] |
| 20 | 1 | NCBInr | gi\|356496575 | 100 | 30620 | 1 | 1 | 1 | 1 | 0,15 | PREDICTED: endochitinase-like [Glycine max] |
| 23 | 1 | NCBInr | gi\|460412845 | 93 | 18183 | 1 | 1 | 1 | 1 | 0,26 | PREDICTED: oleosin 5-like [Solanum lycopersicum] |
| 24 | 1 | NCBInr | gi\|565360525 | 87 | 28477 | 1 | 1 | 1 | 1 | 0,16 | PREDICTED: uncharacterized protein ECU03_1610-like [Solanum tuberosum] |
| 26 | 1 | NCBInr | gi\|30526289 | 81 | 29454 | 1 | 1 | 1 | 1 | 0,15 | 29 kDa chitinase-like thermal hysteresis protein [Solanum dulcamara] |
| 27 | 1 | NCBInr | gi\|147777505 | 79 | 236143 | 1 | 1 | 1 | 1 | 0,02 | hypothetical protein VITISV_027869 [Vitis vinifera] |
| 28 | 1 | NCBInr | gi\|388520197 | 78 | 32109 | 1 | 1 | 1 | 1 | 0,14 | unknown [Lotus japonicus] |
| 30 | 1 | NCBInr | gi\|697185848 | 75 | 15538 | 1 | 1 | 1 | 1 | 0,31 | PREDICTED: oleosin 1-like [Nicotiana tomentosiformis] |
| 31 | 1 | NCBInr | gi\|460373807 | 71 | 32953 | 1 | 1 | 1 | 1 | 0,14 | PREDICTED: putative lactoylglutathione lyase [Solanum lycopersicum] |
| 32 | 1 | NCBInr | gi\|41584319 | 70 | 24573 | 1 | 1 | 1 | 1 | 0,19 | endo-beta-1,3-glucanase [Glycine tabacina] |
| 33 | 1 | NCBInr | gi\|565397210 | 70 | 16932 | 1 | 1 | 1 | 1 | 0,28 | PREDICTED: late embryogenesis abundant protein 1-like [Solanum tuberosum] |
| 34 | 1 | NCBInr | gi\|697103067 | 69 | 36534 | 1 | 1 | 1 | 1 | 0,12 | PREDICTED: peroxidase 3-like [Nicotiana tomentosiformis] |
| 35 | 1 | NCBInr | gi\|697170237 | 66 | 29225 | 1 | 1 | 1 | 1 | 0,15 | PREDICTED: expansin-like A2 isoform X1 [Nicotiana tomentosiformis] |
| 36 | 1 | NCBInr | gi\|22607 | 65 | 29361 | 1 | 1 | 1 | 1 | 0,15 | 14-3-3 protein homologue [Hordeum vulgare subsp. vulgare] |
| 37 | 1 | NCBInr | gi\|460367790 | 63 | 12303 | 1 | 1 | 1 | 1 | 0,4 | PREDICTED: non-specific lipid-transfer protein A-like [Solanum lycopersicum] |
| 38 | 1 | NCBInr | gi\|356519136 | 61 | 42869 | 1 | 1 | 1 | 1 | 0,1 | PREDICTED: serpin-ZX-like [Glycine max] |
| 39 | 1 | NCBInr | gi\|460386382 | 60 | 43211 | 1 | 1 | 1 | 1 | 0,1 | PREDICTED: probable fructose-bisphosphate aldolase 3, chloroplastic [Solanum lycopersicum] |
| 40 | 1 | NCBInr | gi\|460393933 | 60 | 41939 | 1 | 1 | 1 | 1 | 0,11 | PREDICTED: chitotriosidase-1 [Solanum lycopersicum] |
| 41 | 1 | NCBInr | gi\|596224101 | 60 | 35011 | 2 | 2 | 1 | 1 | 0,13 | hypothetical protein PRUPE_ppa015818mg [Prunus persica] |
| 42 | 1 | NCBInr | gi\|255554527 | 60 | 34062 | 1 | 1 | 1 | 1 | 0,13 | inorganic pyrophosphatase, putative [Ricinus communis] |
| 43 | 1 | NCBInr | gi\|661882206 | 59 | 42830 | 1 | 1 | 1 | 1 | 0,1 | unnamed protein product [Coffea canephora] |
| 44 | 1 | NCBInr | gi\|119905 | 59 | 40454 | 1 | 1 | 1 | 1 | 0,11 | RecName: Full=Ferredoxin--NADP reductase, leaf isozyme, chloroplastic; Short=FNR; Flags: Precursor [Pisum sativum] |
| 45 | 1 | NCBInr | gi\|166165272 | 58 | 17699 | 1 | 1 | 1 | 1 | 0,27 | cystatin [Solanum lycopersicum] |
| 46 | 1 | NCBInr | gi\|121982 | 58 | 16060 | 1 | 1 | 1 | 1 | 0,29 | RecName: Full=Histone H2A.2.2 [Triticum aestivum] |
| 47 | 1 | NCBInr | gi\|659098604 | 57 | 18645 | 1 | 1 | 1 | 1 | 0,25 | PREDICTED: uncharacterized protein LOC103491886 [Cucumis melo] |
| 48 | 1 | NCBInr | gi\|1616628 | 55 | 20764 | 1 | 1 | 1 | 1 | 0,22 | sts15 [Solanum tuberosum] |
| 49 | 1 | NCBInr | gi\|593784427 | 54 | 42506 | 1 | 1 | 1 | 1 | 0,1 | hypothetical protein PHAVU_003G186200g [Phaseolus vulgaris] |
| 50 | 1 | NCBInr | gi\|356495935 | 54 | 103561 | 1 | 1 | 1 | 1 | 0,04 | PREDICTED: alpha-xylosidase 1-like [Glycine max] |
| 51 | 1 | NCBInr | gi\|55296956 | 53 | 56025 | 1 | 1 | 1 | 1 | 0,08 | hypothetical protein [Oryza sativa Japonica Group] |
| 52 | 1 | NCBInr | gi\|566213650 | 52 | 34722 | 1 | 1 | 1 | 1 | 0,13 | hypothetical protein POPTR_0018s01440g [Populus trichocarpa] |
| 53 | 1 | NCBInr | gi\|723456942 | 51 | 22690 | 1 | 1 | 1 | 1 | 0,2 | hypothetical chloroplast RF4 (chloroplast) [Neocystis brevis] |
| 54 | 1 | NCBInr | gi\|20022 | 51 | 21942 | 1 | 1 | 1 | 1 | 0,21 | ribosomal protein S6 [Nicotiana tabacum] |
| 55 | 1 | NCBInr | gi\|470132429 | 51 | 31022 | 1 | 1 | 1 | 1 | 0,14 | PREDICTED: phosphoenolpyruvate carboxylase kinase 1-like isoform 2 [Fragaria vesca subsp. vesca] |
| 56 | 1 | NCBInr | gi\|661894397 | 51 | 17694 | 1 | 1 | 1 | 1 | 0,27 | unnamed protein product [Coffea canephora] |
| 57 | 1 | NCBInr | gi\|545361237 | 51 | 23745 | 1 | 1 | 1 | 1 | 0,19 | hypothetical protein COCSUDRAFT_56597 [Coccomyxa subellipsoidea C-169] |
| 58 | 1 | NCBInr | gi\|545370097 | 50 | 93458 | 2 | 2 | 1 | 1 | 0,05 | hypothetical protein COCSUDRAFT_65136 [Coccomyxa subellipsoidea C-169] |
| 59 | 1 | NCBInr | gi\|123536 | 49 | 8405 | 1 | 1 | 1 | 1 | 0,62 | RecName: Full=Class I heat shock protein, partial [Glycine max] |
| 60 | 1 | NCBInr | gi\|159467815 | 49 | 42969 | 1 | 1 | 1 | 1 | 0,1 | ubiquitin family protein [Chlamydomonas reinhardtii] |
| 61 | 1 | NCBInr | gi\|658038245 | 49 | 55030 | 1 | 1 | 1 | 1 | 0,08 | PREDICTED: probable disease resistance protein At5g66900 [Malus domestica] |
| 62 | 1 | NCBInr | gi\|674244918 | 49 | 571656 | 1 | 1 | 1 | 1 | 0,01 | hypothetical protein AALP_AA3G015100 [Arabis alpina] |
| 63 | 1 | NCBInr | gi\|413937215 | 48 | 73254 | 1 | 1 | 1 | 1 | 0,06 | putative protein of unknown function (DUF668) domain family protein [Zea mays] |
| 64 | 1 | NCBInr | gi\|147843231 | 48 | 89118 | 1 | 1 | 1 | 1 | 0,05 | hypothetical protein VITISV_019389 [Vitis vinifera] |
| 65 | 1 | NCBInr | gi\|698511377 | 48 | 19529 | 1 | 1 | 1 | 1 | 0,24 | PREDICTED: ATP-dependent RNA helicase SUPV3L1, mitochondrial-like [Nicotiana sylvestris] |
| 66 | 1 | NCBInr | gi\|357110912 | 48 | 86292 | 1 | 1 | 1 | 1 | 0,05 | PREDICTED: cell division control protein 48 homolog C [Brachypodium distachyon] |
| 67 | 1 | NCBInr | gi\|294461233 | 47 | 28036 | 1 | 1 | 1 | 1 | 0,16 | unknown [Picea sitchensis] |
| 68 | 1 | NCBInr | gi\|629113167 | 47 | 126348 | 1 | 1 | 1 | 1 | 0,03 | hypothetical protein EUGRSUZ_D02334 [Eucalyptus grandis] |
| 69 | 1 | NCBInr | gi\|700253140 | 47 | 157100 | 1 | 1 | 1 | 1 | 0,03 | MSH1, partial [Fragaria vesca] |
| 70 | 1 | NCBInr | gi\|147766466 | 47 | 30370 | 1 | 1 | 1 | 1 | 0,15 | hypothetical protein VITISV_034331 [Vitis vinifera] |
| 71 | 1 | NCBInr | gi\|15226197 | 47 | 79284 | 1 | 1 | 1 | 1 | 0,05 | leucine-rich repeat transmembrane protein kinase-like protein [Arabidopsis thaliana] |
| 72 | 1 | NCBInr | gi\|259161 | 47 | 3007 | 1 | 1 | 1 | 1 | 2,5 | AP24=osmotin homolog {N-terminal} [tomato, Peptide Partial, 27 aa] |
| 73 | 1 | NCBInr | gi\|4455214 | 46 | 56079 | 1 | 1 | 1 | 1 | 0,08 | putative dihydrolipoamide succinyltransferase [Arabidopsis thaliana] |
| 74 | 1 | NCBInr | gi\|2244755 | 46 | 57107 | 1 | 1 | 1 | 1 | 0,08 | hypothetical protein [Arabidopsis thaliana] |
| 75 | 1 | NCBInr | gi\|629085054 | 45 | 33650 | 1 | 1 | 1 | 1 | 0,13 | hypothetical protein EUGRSUZ_J00948 [Eucalyptus grandis] |
| 76 | 1 | NCBInr | gi\|674873875 | 45 | 96410 | 1 | 1 | 1 | 1 | 0,04 | BnaC07g51120D [Brassica napus] |
| 77 | 1 | NCBInr | gi\|302788983 | 45 | 34013 | 1 | 1 | 1 | 1 | 0,13 | hypothetical protein SELMODRAFT_443146 [Selaginella moellendorffii] |
| 78 | 1 | NCBInr | gi\|971280 | 44 | 24298 | 1 | 1 | 1 | 1 | 0,19 | RAB24 protein [Oryza sativa (japonica cultivar-group)] |
| 79 | 1 | NCBInr | gi\|227438221 | 44 | 104329 | 1 | 1 | 1 | 1 | 0,04 | disease resistance protein [Brassica rapa subsp. pekinensis] |
| 80 | 1 | NCBInr | gi\|167997063 | 44 | 26626 | 1 | 1 | 1 | 1 | 0,17 | predicted protein [Physcomitrella patens] |
| 81 | 1 | NCBInr | gi\|565498284 | 43 | 95218 | 1 | 1 | 1 | 1 | 0,05 | hypothetical protein CARUB_v10008321mg, partial [Capsella rubella] |
| 82 | 1 | NCBInr | gi\|723698582 | 43 | 50272 | 1 | 1 | 1 | 1 | 0,09 | PREDICTED: LOW QUALITY PROTEIN: probable leucine-rich repeat receptor-like protein kinase At1g35710 [Solanum lycopersicum] |
| 6 | 1 | NCBInr | gi\|124701 | 175 | 70338 | 2 | 2 | 1 | 1 | 0,06 | RecName: Full=Acid beta-fructofuranosidase; AltName: Full=Acid invertase; Short=AI; AltName: Full=Acid sucrose hydrolase; AltName: Full=Vacuolar invertase; Flags: Precursor [Solanum lycopersicum] |
| 19 | 1 | NCBInr | gi\|449468680 | 100 | 57134 | 1 | 1 | 1 | 1 | 0,08 | PREDICTED: 11S globulin subunit beta-like [Cucumis sativus] |
| 21 | 3 | NCBInr | gi\|116789891 | 58 | 15054 | 1 | 1 | 1 | 1 | 0,32 | unknown [Picea sitchensis] |
| 22 | 1 | NCBInr | gi\|723696964 | 96 | 17196 | 1 | 1 | 1 | 1 | 0,27 | PREDICTED: MLP-like protein 423 [Solanum lycopersicum] |
| 24 | 1 | NCBInr | gi\|565369785 | 92 | 24426 | 1 | 1 | 1 | 1 | 0,19 | PREDICTED: probable serine protease inhibitor 6-like [Solanum tuberosum] |
| 25 | 1 | NCBInr | gi\|134616 | 91 | 15340 | 1 | 1 | 1 | 1 | 0,31 | RecName: Full=Superoxide dismutase [Nicotiana plumbaginifolia] |
| 26 | 1 | NCBInr | gi\|89275333 | 89 | 8746 | 1 | 1 | 1 | 1 | 0,59 | EM1 [Phoenix dactylifera] |
| 27 | 1 | NCBInr | gi\|657974265 | 89 | 22959 | 2 | 2 | 1 | 1 | 0,2 | PREDICTED: 22.0 kDa class IV heat shock protein-like [Malus domestica] |
| 30 | 1 | NCBInr | gi\|729405394 | 85 | 53184 | 1 | 1 | 1 | 1 | 0,08 | PREDICTED: cruciferin PGCRURSE5 [Tarenaya hassleriana] |
| 32 | 1 | NCBInr | gi\|3986695 | 83 | 15280 | 1 | 1 | 1 | 1 | 0,31 | ribosomal protein L12 [Cichorium intybus] |
| 33 | 1 | NCBInr | gi\|604348300 | 82 | 53260 | 2 | 2 | 1 | 1 | 0,08 | hypothetical protein MIMGU_mgv1a005708mg [Erythranthe guttata] |
| 35 | 1 | NCBInr | gi\|30526289 | 79 | 29454 | 1 | 1 | 1 | 1 | 0,15 | 29 kDa chitinase-like thermal hysteresis protein [Solanum dulcamara] |
| 36 | 1 | NCBInr | gi\|697185848 | 78 | 15538 | 1 | 1 | 1 | 1 | 0,31 | PREDICTED: oleosin 1-like [Nicotiana tomentosiformis] |
| 37 | 1 | NCBInr | gi\|302771612 | 77 | 89174 | 2 | 2 | 1 | 1 | 0,05 | hypothetical protein SELMODRAFT_410159 [Selaginella moellendorffii] |
| 38 | 1 | NCBInr | gi\|565397210 | 75 | 16932 | 1 | 1 | 1 | 1 | 0,28 | PREDICTED: late embryogenesis abundant protein 1-like [Solanum tuberosum] |
| 39 | 1 | NCBInr | gi\|568214545 | 75 | 17209 | 1 | 1 | 1 | 1 | 0,27 | 60S ribosomal protein L23a-like [Solanum tuberosum] |
| 40 | 1 | NCBInr | gi\|460384740 | 73 | 103520 | 1 | 1 | 1 | 1 | 0,04 | PREDICTED: uncharacterized protein LOC101252078 [Solanum lycopersicum] |
| 41 | 1 | NCBInr | gi\|14423789 | 73 | 7697 | 1 | 1 | 1 | 1 | 0,7 | RecName: Full=Non-specific lipid-transfer protein 2; Short=LTP 2 [Prunus armeniaca] |
| 43 | 1 | NCBInr | gi\|168046576 | 70 | 18128 | 1 | 1 | 1 | 1 | 0,26 | predicted protein [Physcomitrella patens] |
| 45 | 1 | NCBInr | gi\|590695935 | 69 | 83992 | 1 | 1 | 1 | 1 | 0,05 | Ankyrin repeat [Theobroma cacao] |
| 47 | 1 | NCBInr | gi\|527191418 | 68 | 53784 | 1 | 1 | 1 | 1 | 0,08 | hypothetical protein M569_12535, partial [Genlisea aurea] |
| 48 | 1 | NCBInr | gi\|46091271 | 67 | 69064 | 1 | 1 | 1 | 1 | 0,06 | exo-1,3-beta-glucanase [Lilium longiflorum] |
| 49 | 1 | NCBInr | gi\|4455214 | 67 | 56079 | 1 | 1 | 1 | 1 | 0,08 | putative dihydrolipoamide succinyltransferase [Arabidopsis thaliana] |
| 50 | 1 | NCBInr | gi\|674244918 | 66 | 571656 | 1 | 1 | 1 | 1 | 0,01 | hypothetical protein AALP_AA3G015100 [Arabis alpina] |
| 51 | 1 | NCBInr | gi\|695023069 | 62 | 33515 | 1 | 1 | 1 | 1 | 0,13 | PREDICTED: probable plastid-lipid-associated protein 2, chloroplastic [Musa acuminata subsp. malaccensis] |
| 52 | 1 | NCBInr | gi\|15242241 | 58 | 23079 | 1 | 1 | 1 | 1 | 0,2 | 40S ribosomal protein S9-1 [Arabidopsis thaliana] |
| 53 | 1 | NCBInr | gi\|703139150 | 57 | 48044 | 1 | 1 | 1 | 1 | 0,09 | hypothetical protein L484_013003 [Morus notabilis] |
| 54 | 1 | NCBInr | gi\|132270 | 57 | 14713 | 1 | 1 | 1 | 1 | 0,32 | RecName: Full=Rubber elongation factor protein; Short=REF; AltName: Allergen=Hev b 1 [Hevea brasiliensis] |
| 55 | 1 | NCBInr | gi\|565375983 | 57 | 25139 | 1 | 1 | 1 | 1 | 0,18 | PREDICTED: germin-like protein subfamily 1 member 7-like [Solanum tuberosum] |
| 56 | 1 | NCBInr | gi\|693501245 | 57 | 58247 | 1 | 1 | 1 | 1 | 0,08 | unnamed product [Ostreococcus tauri] |
| 57 | 1 | NCBInr | gi\|302857413 | 57 | 6231 | 1 | 1 | 1 | 1 | 0,9 | hypothetical protein VOLCADRAFT_71920 [Volvox carteri f. nagariensis] |
| 58 | 1 | NCBInr | gi\|227438221 | 57 | 104329 | 1 | 1 | 1 | 1 | 0,04 | disease resistance protein [Brassica rapa subsp. pekinensis] |
| 59 | 1 | NCBInr | gi\|672143638 | 56 | 87707 | 1 | 1 | 1 | 1 | 0,05 | PREDICTED: uncharacterized protein LOC103711369 [Phoenix dactylifera] |
| 60 | 1 | NCBInr | gi\|168044085 | 56 | 106084 | 1 | 1 | 1 | 1 | 0,04 | predicted protein [Physcomitrella patens] |
| 61 | 1 | NCBInr | gi\|1616628 | 56 | 20764 | 1 | 1 | 1 | 1 | 0,22 | sts15 [Solanum tuberosum] |
| 62 | 1 | NCBInr | gi\|123545 | 55 | 16868 | 1 | 1 | 1 | 1 | 0,28 | RecName: Full=16.9 kDa class I heat shock protein 1; AltName: Full=HSP 16.9; AltName: Full=Heat shock protein 16.9A; AltName: Full=Heat shock protein 17; AltName: Full=Low molecular weight heat shock protein [Triticum aestivum] |
| 63 | 1 | NCBInr | gi\|147782453 | 55 | 81175 | 1 | 1 | 1 | 1 | 0,05 | hypothetical protein VITISV_006352 [Vitis vinifera] |
| 66 | 1 | NCBInr | gi\|566199009 | 54 | 37929 | 1 | 1 | 1 | 1 | 0,12 | hypothetical protein POPTR_0013s02480g [Populus trichocarpa] |
| 67 | 1 | NCBInr | gi\|326531426 | 53 | 115601 | 1 | 1 | 1 | 1 | 0,04 | predicted protein [Hordeum vulgare subsp. vulgare] |
| 68 | 1 | NCBInr | gi\|1438518 | 53 | 14232 | 1 | 1 | 1 | 1 | 0,34 | dhn1 [Solanum commersonii] |
| 70 | 1 | NCBInr | gi\|1076411 | 53 | 1086 | 1 | 1 | 1 | 1 | 15,8 | seed storage protein beta-chain 6 - Arabidopsis thaliana (fragment) |
| 71 | 1 | NCBInr | gi\|475569069 | 53 | 30636 | 1 | 1 | 1 | 1 | 0,15 | hypothetical protein F775_04563 [Aegilops tauschii] |
| 72 | 1 | NCBInr | gi\|566177402 | 53 | 20861 | 1 | 1 | 1 | 1 | 0,22 | hypothetical protein POPTR_0006s21250g [Populus trichocarpa] |
| 73 | 1 | NCBInr | gi\|124230 | 53 | 17876 | 1 | 1 | 1 | 1 | 0,26 | RecName: Full=Eukaryotic translation initiation factor 5A-1; Short=eIF-5A-1; AltName: Full=eIF-4D [Medicago sativa] |
| 74 | 1 | NCBInr | gi\|727437173 | 52 | 50639 | 1 | 1 | 1 | 1 | 0,09 | PREDICTED: leucine-rich repeat receptor-like serine/threonine-protein kinase BAM1 [Camelina sativa] |
| 75 | 1 | NCBInr | gi\|697173573 | 52 | 96286 | 1 | 1 | 1 | 1 | 0,05 | PREDICTED: vicilin-like antimicrobial peptides 2-3 isoform X1 [Nicotiana tomentosiformis] |
| 76 | 1 | NCBInr | gi\|596224101 | 52 | 35011 | 1 | 1 | 1 | 1 | 0,13 | hypothetical protein PRUPE_ppa015818mg [Prunus persica] |
| 77 | 1 | NCBInr | gi\|18396352 | 52 | 25276 | 1 | 1 | 1 | 1 | 0,18 | vacuolar protein sorting-associated protein 2-1 [Arabidopsis thaliana] |
| 78 | 1 | NCBInr | gi\|697120734 | 51 | 17868 | 1 | 1 | 1 | 1 | 0,26 | PREDICTED: uncharacterized protein LOC104107282 [Nicotiana tomentosiformis] |
| 79 | 1 | NCBInr | gi\|295792020 | 51 | 27630 | 1 | 1 | 1 | 1 | 0,16 | NADH-plastoquinone oxidoreductase subunit 2, partial (chloroplast) [Lygodium japonicum] |
| 80 | 1 | NCBInr | gi\|3299896 | 51 | 81089 | 1 | 1 | 1 | 1 | 0,05 | beta-galactosidase [Solanum lycopersicum] |
| 81 | 1 | NCBInr | gi\|604298237 | 51 | 28399 | 1 | 1 | 1 | 1 | 0,16 | hypothetical protein MIMGU_mgv1a0236971mg, partial [Erythranthe guttata] |
| 82 | 1 | NCBInr | gi\|661894397 | 51 | 17694 | 1 | 1 | 1 | 1 | 0,27 | unnamed protein product [Coffea canephora] |
| 83 | 1 | NCBInr | gi\|732905 | 51 | 28271 | 1 | 1 | 1 | 1 | 0,16 | orf [Pisum sativum] |
| 84 | 1 | NCBInr | gi\|1076408 | 51 | 2124 | 1 | 1 | 1 | 1 | 4,45 | seed storage protein beta-chain 3 - Arabidopsis thaliana (fragment) |
| 85 | 1 | NCBInr | gi\|259161 | 50 | 3007 | 1 | 1 | 1 | 1 | 2,5 | AP24=osmotin homolog {N-terminal} [tomato, Peptide Partial, 27 aa] |
| 86 | 1 | NCBInr | gi\|15239085 | 50 | 84825 | 1 | 1 | 1 | 1 | 0,05 | pentatricopeptide repeat-containing protein [Arabidopsis thaliana] |
| 87 | 1 | NCBInr | gi\|514783407 | 47 | 62278 | 1 | 1 | 1 | 1 | 0,07 | PREDICTED: type I inositol 1,4,5-trisphosphate 5-phosphatase CVP2-like isoform X3 [Setaria italica] |
| 88 | 1 | NCBInr | gi\|147791054 | 47 | 174993 | 1 | 1 | 1 | 1 | 0,02 | hypothetical protein VITISV_003625 [Vitis vinifera] |
| 89 | 1 | NCBInr | gi\|460410827 | 47 | 39793 | 1 | 1 | 1 | 1 | 0,11 | PREDICTED: GDSL esterase/lipase At1g71250 [Solanum lycopersicum] |
| 90 | 1 | NCBInr | gi\|55296895 | 46 | 21876 | 1 | 1 | 1 | 1 | 0,21 | hypothetical protein [Oryza sativa Japonica Group] |
| 91 | 1 | NCBInr | gi\|695003146 | 46 | 74996 | 1 | 1 | 1 | 1 | 0,06 | PREDICTED: NAC domain-containing protein 78-like isoform X1 [Musa acuminata subsp. malaccensis] |
| 93 | 1 | NCBInr | gi\|15226197 | 45 | 79284 | 1 | 1 | 1 | 1 | 0,06 | leucine-rich repeat transmembrane protein kinase-like protein [Arabidopsis thaliana] |
| 94 | 1 | NCBInr | gi\|294461233 | 45 | 28036 | 1 | 1 | 1 | 1 | 0,16 | unknown [Picea sitchensis] |
| 95 | 1 | NCBInr | gi\|470116029 | 44 | 58959 | 1 | 1 | 1 | 1 | 0,07 | PREDICTED: secologanin synthase-like [Fragaria vesca subsp. vesca] |
| 96 | 1 | NCBInr | gi\|743796727 | 44 | 51710 | 1 | 1 | 1 | 1 | 0,09 | PREDICTED: serine/threonine-protein kinase BLUS1-like [Populus euphratica] |
| 97 | 1 | NCBInr | gi\|16303740 | 43 | 57682 | 1 | 1 | 1 | 1 | 0,08 | beta-glucosidase isozyme 2 precursor [Oryza sativa Japonica Group] |
| 98 | 1 | NCBInr | gi\|672167438 | 43 | 35309 | 1 | 1 | 1 | 1 | 0,13 | PREDICTED: LOW QUALITY PROTEIN: probable ubiquitin-like-specific protease 2A [Phoenix dactylifera] |
| 5 | 1 | NCBInr | gi\|460404101 | 89 | 54717 | 1 | 1 | 1 | 1 | 0,08 | PREDICTED: 11S globulin seed storage protein 2-like [Solanum lycopersicum] |
| 6 | 1 | NCBInr | gi\|729405394 | 84 | 53184 | 1 | 1 | 1 | 1 | 0,08 | PREDICTED: cruciferin PGCRURSE5 [Tarenaya hassleriana] |
| 7 | 1 | NCBInr | gi\|566223266 | 78 | 56368 | 1 | 1 | 1 | 1 | 0,08 | hypothetical protein POPTR_0019s01840g [Populus trichocarpa] |
| 8 | 1 | NCBInr | gi\|227438221 | 71 | 104329 | 1 | 1 | 1 | 1 | 0,04 | disease resistance protein [Brassica rapa subsp. pekinensis] |
| 9 | 1 | NCBInr | gi\|15239136 | 71 | 13272 | 1 | 1 | 1 | 1 | 0,36 | thioredoxin H3 [Arabidopsis thaliana] |
| 10 | 1 | NCBInr | gi\|527191418 | 69 | 53784 | 1 | 1 | 1 | 1 | 0,08 | hypothetical protein M569_12535, partial [Genlisea aurea] |
| 11 | 1 | NCBInr | gi\|527205827 | 68 | 76721 | 1 | 1 | 1 | 1 | 0,06 | hypothetical protein M569_03185 [Genlisea aurea] |
| 12 | 1 | NCBInr | gi\|693501245 | 67 | 58247 | 1 | 1 | 1 | 1 | 0,08 | unnamed product [Ostreococcus tauri] |
| 13 | 1 | NCBInr | gi\|565450543 | 67 | 42944 | 1 | 1 | 1 | 1 | 0,1 | hypothetical protein CARUB_v10007243mg, partial [Capsella rubella] |
| 14 | 1 | NCBInr | gi\|302771612 | 66 | 89174 | 1 | 1 | 1 | 1 | 0,05 | hypothetical protein SELMODRAFT_410159 [Selaginella moellendorffii] |
| 15 | 1 | NCBInr | gi\|604343782 | 63 | 13662 | 1 | 1 | 1 | 1 | 0,35 | hypothetical protein MIMGU_mgv1a016253mg [Erythranthe guttata] |
| 16 | 1 | NCBInr | gi\|657985907 | 61 | 133673 | 2 | 2 | 1 | 1 | 0,03 | PREDICTED: putative disease resistance protein At3g14460 [Malus domestica] |
| 17 | 1 | NCBInr | gi\|159463262 | 60 | 118165 | 1 | 1 | 1 | 1 | 0,04 | predicted protein [Chlamydomonas reinhardtii] |
| 18 | 1 | NCBInr | gi\|604312292 | 59 | 49277 | 1 | 1 | 1 | 1 | 0,09 | hypothetical protein MIMGU_mgv1a024803mg, partial [Erythranthe guttata] |
| 19 | 1 | NCBInr | gi\|674244918 | 59 | 571656 | 1 | 1 | 1 | 1 | 0,01 | hypothetical protein AALP_AA3G015100 [Arabis alpina] |
| 20 | 1 | NCBInr | gi\|719968548 | 59 | 22760 | 1 | 1 | 1 | 1 | 0,2 | PREDICTED: vicilin-like antimicrobial peptides 2-2 [Nelumbo nucifera] |
| 21 | 1 | NCBInr | gi\|222636648 | 57 | 38910 | 1 | 1 | 1 | 1 | 0,11 | hypothetical protein OsJ_23512 [Oryza sativa Japonica Group] |
| 22 | 1 | NCBInr | gi\|545370097 | 56 | 93458 | 2 | 2 | 1 | 1 | 0,05 | hypothetical protein COCSUDRAFT_65136 [Coccomyxa subellipsoidea C-169] |
| 23 | 1 | NCBInr | gi\|1616628 | 55 | 20764 | 1 | 1 | 1 | 1 | 0,22 | sts15 [Solanum tuberosum] |
| 24 | 1 | NCBInr | gi\|94502538 | 55 | 205456 | 2 | 2 | 1 | 1 | 0,02 | hypothetical chloroplast RF1 (chloroplast) [Helianthus annuus] |
| 25 | 1 | NCBInr | gi\|115444361 | 54 | 44689 | 1 | 1 | 1 | 1 | 0,1 | Os02g0158900 [Oryza sativa Japonica Group] |
| 26 | 1 | NCBInr | gi\|84579412 | 54 | 64534 | 1 | 1 | 1 | 1 | 0,07 | Lactuca sativa 9-cis-epoxycarotenoid dioxygenase 4 |
| 27 | 1 | NCBInr | gi\|2055384 | 54 | 23712 | 1 | 1 | 1 | 1 | 0,19 | cold-stress inducible protein [Solanum tuberosum] |
| 28 | 1 | NCBInr | gi\|593784427 | 53 | 42506 | 1 | 1 | 1 | 1 | 0,1 | hypothetical protein PHAVU_003G186200g [Phaseolus vulgaris] |
| 29 | 1 | NCBInr | gi\|116268418 | 53 | 54033 | 1 | 1 | 1 | 1 | 0,08 | hypothetical protein [Prunus persica] |
| 30 | 1 | NCBInr | gi\|12323328 | 53 | 112045 | 1 | 1 | 1 | 1 | 0,04 | unknown protein; 69131-60853 [Arabidopsis thaliana] |
| 31 | 1 | NCBInr | gi\|295792020 | 51 | 27630 | 1 | 1 | 1 | 1 | 0,16 | NADH-plastoquinone oxidoreductase subunit 2, partial (chloroplast) [Lygodium japonicum] |
| 32 | 1 | NCBInr | gi\|657945465 | 51 | 145624 | 1 | 1 | 1 | 1 | 0,03 | PREDICTED: dentin sialophosphoprotein-like isoform X1 [Malus domestica] |
| 33 | 1 | NCBInr | gi\|470132429 | 51 | 31022 | 1 | 1 | 1 | 1 | 0,15 | PREDICTED: phosphoenolpyruvate carboxylase kinase 1-like isoform 2 [Fragaria vesca subsp. vesca] |
| 34 | 1 | NCBInr | gi\|15218496 | 51 | 35381 | 1 | 1 | 1 | 1 | 0,13 | uncharacterized protein [Arabidopsis thaliana] |
| 35 | 1 | NCBInr | gi\|147819109 | 51 | 99818 | 1 | 1 | 1 | 1 | 0,04 | hypothetical protein VITISV_014193 [Vitis vinifera] |
| 36 | 1 | NCBInr | gi\|567150070 | 50 | 75927 | 1 | 1 | 1 | 1 | 0,06 | hypothetical protein EUTSA_v10006991mg [Eutrema salsugineum] |
| 37 | 1 | NCBInr | gi\|15241031 | 50 | 41031 | 1 | 1 | 1 | 1 | 0,11 | replication factor C subunit 3 [Arabidopsis thaliana] |
| 38 | 1 | NCBInr | gi\|590665414 | 49 | 72681 | 1 | 1 | 1 | 1 | 0,06 | Uncharacterized protein TCM_012637 [Theobroma cacao] |
| 39 | 1 | NCBInr | gi\|15226197 | 49 | 79284 | 1 | 1 | 1 | 1 | 0,05 | leucine-rich repeat transmembrane protein kinase-like protein [Arabidopsis thaliana] |
| 40 | 1 | NCBInr | gi\|297806141 | 49 | 40142 | 1 | 1 | 1 | 1 | 0,11 | CYCD7_1 [Arabidopsis lyrata subsp. lyrata] |
| 41 | 1 | NCBInr | gi\|695065538 | 49 | 116531 | 1 | 1 | 1 | 1 | 0,04 | PREDICTED: calcium-transporting ATPase 1, endoplasmic reticulum-type-like [Musa acuminata subsp. malaccensis] |
| 42 | 1 | NCBInr | gi\|658031990 | 49 | 75297 | 1 | 1 | 1 | 1 | 0,06 | PREDICTED: uncharacterized protein LOC103414907 [Malus domestica] |
| 43 | 1 | NCBInr | gi\|674873875 | 48 | 96410 | 1 | 1 | 1 | 1 | 0,05 | BnaC07g51120D [Brassica napus] |
| 44 | 1 | NCBInr | gi\|147843231 | 48 | 89118 | 1 | 1 | 1 | 1 | 0,05 | hypothetical protein VITISV_019389 [Vitis vinifera] |
| 45 | 1 | NCBInr | gi\|629113167 | 48 | 126348 | 2 | 1 | 1 | 1 | 0,03 | hypothetical protein EUGRSUZ_D02334 [Eucalyptus grandis] |
| 46 | 1 | NCBInr | gi\|565366241 | 48 | 55441 | 1 | 1 | 1 | 1 | 0,08 | PREDICTED: uncharacterized protein LOC102600818 [Solanum tuberosum] |
| 47 | 1 | NCBInr | gi\|604335134 | 48 | 31547 | 1 | 1 | 1 | 1 | 0,14 | hypothetical protein MIMGU_mgv1a010985mg [Erythranthe guttata] |
| 48 | 1 | NCBInr | gi\|294461233 | 48 | 28036 | 1 | 1 | 1 | 1 | 0,16 | unknown [Picea sitchensis] |
| 49 | 1 | NCBInr | gi\|8249041 | 48 | 47670 | 1 | 1 | 1 | 1 | 0,09 | phenylalanine ammonia-lyase [Betula pendula] |
| 50 | 1 | NCBInr | gi\|674895785 | 47 | 30918 | 1 | 1 | 1 | 1 | 0,15 | BnaA04g26030D [Brassica napus] |
| 51 | 1 | NCBInr | gi\|723456942 | 47 | 22690 | 1 | 1 | 1 | 1 | 0,2 | hypothetical chloroplast RF4 (chloroplast) [Neocystis brevis] |
| 52 | 1 | NCBInr | gi\|470124256 | 47 | 147681 | 1 | 1 | 1 | 1 | 0,03 | PREDICTED: condensin-2 complex subunit D3-like [Fragaria vesca subsp. vesca] |
| 53 | 1 | NCBInr | gi\|595800832 | 47 | 69195 | 1 | 1 | 1 | 1 | 0,06 | hypothetical protein PRUPE_ppa002913mg [Prunus persica] |
| 54 | 1 | NCBInr | gi\|566213650 | 47 | 34722 | 1 | 1 | 1 | 1 | 0,13 | hypothetical protein POPTR_0018s01440g [Populus trichocarpa] |
| 55 | 1 | NCBInr | gi\|604348300 | 46 | 53260 | 1 | 1 | 1 | 1 | 0,08 | hypothetical protein MIMGU_mgv1a005708mg [Erythranthe guttata] |
| 56 | 1 | NCBInr | gi\|308803310 | 46 | 27367 | 1 | 1 | 1 | 1 | 0,17 | unnamed protein product [Ostreococcus tauri] |
| 57 | 1 | NCBInr | gi\|514783407 | 46 | 62278 | 1 | 1 | 1 | 1 | 0,07 | PREDICTED: type I inositol 1,4,5-trisphosphate 5-phosphatase CVP2-like isoform X3 [Setaria italica] |
| 58 | 1 | NCBInr | gi\|255082530 | 46 | 111061 | 1 | 1 | 1 | 1 | 0,04 | predicted protein [Micromonas sp. RCC299] |
| 59 | 1 | NCBInr | gi\|727433313 | 46 | 40484 | 1 | 1 | 1 | 1 | 0,11 | PREDICTED: arogenate dehydrogenase 2, chloroplastic-like [Camelina sativa] |
| 60 | 1 | NCBInr | gi\|162463394 | 45 | 97905 | 1 | 1 | 1 | 1 | 0,04 | lipoxygenase6 [Zea mays] |
| 61 | 1 | NCBInr | gi\|672189919 | 44 | 65337 | 1 | 1 | 1 | 1 | 0,07 | PREDICTED: globulin-1 S allele [Phoenix dactylifera] |
| 62 | 1 | NCBInr | gi\|255557524 | 44 | 48348 | 1 | 1 | 1 | 1 | 0,09 | protein binding protein, putative [Ricinus communis] |
| 63 | 1 | NCBInr | gi\|702262200 | 44 | 37297 | 1 | 1 | 1 | 1 | 0,12 | PREDICTED: uncharacterized protein At4g15545-like [Eucalyptus grandis] |
| 64 | 1 | NCBInr | gi\|167997063 | 44 | 26626 | 1 | 1 | 1 | 1 | 0,17 | predicted protein [Physcomitrella patens] |
| 12 | 1 | NCBInr | gi\|565360525 | 105 | 28477 | 1 | 1 | 1 | 1 | 0,16 | PREDICTED: uncharacterized protein ECU03_1610-like [Solanum tuberosum] |
| 13 | 1 | NCBInr | gi\|698503706 | 99 | 32454 | 2 | 2 | 1 | 1 | 0,14 | PREDICTED: ABA-inducible protein PHV A1-like [Nicotiana sylvestris] |
| 14 | 1 | NCBInr | gi\|729405394 | 89 | 53184 | 1 | 1 | 1 | 1 | 0,08 | PREDICTED: cruciferin PGCRURSE5 [Tarenaya hassleriana] |
| 16 | 1 | NCBInr | gi\|460367790 | 82 | 12303 | 1 | 1 | 1 | 1 | 0,4 | PREDICTED: non-specific lipid-transfer protein A-like [Solanum lycopersicum] |
| 18 | 1 | NCBInr | gi\|460402809 | 76 | 46635 | 1 | 1 | 1 | 1 | 0,09 | PREDICTED: embryonic protein DC-8 [Solanum lycopersicum] |
| 19 | 1 | NCBInr | gi\|4455214 | 72 | 56079 | 1 | 1 | 1 | 1 | 0,08 | putative dihydrolipoamide succinyltransferase [Arabidopsis thaliana] |
| 20 | 1 | NCBInr | gi\|527191418 | 70 | 53784 | 1 | 1 | 1 | 1 | 0,08 | hypothetical protein M569_12535, partial [Genlisea aurea] |
| 21 | 1 | NCBInr | gi\|565397210 | 70 | 16932 | 1 | 1 | 1 | 1 | 0,28 | PREDICTED: late embryogenesis abundant protein 1-like [Solanum tuberosum] |
| 22 | 1 | NCBInr | gi\|674244918 | 66 | 571656 | 1 | 1 | 1 | 1 | 0,01 | hypothetical protein AALP_AA3G015100 [Arabis alpina] |
| 23 | 1 | NCBInr | gi\|302771612 | 62 | 89174 | 1 | 1 | 1 | 1 | 0,05 | hypothetical protein SELMODRAFT_410159 [Selaginella moellendorffii] |
| 24 | 1 | NCBInr | gi\|657985907 | 62 | 133673 | 1 | 1 | 1 | 1 | 0,03 | PREDICTED: putative disease resistance protein At3g14460 [Malus domestica] |
| 25 | 1 | NCBInr | gi\|693501245 | 62 | 58247 | 1 | 1 | 1 | 1 | 0,08 | unnamed product [Ostreococcus tauri] |
| 26 | 1 | NCBInr | gi\|719968548 | 59 | 22760 | 1 | 1 | 1 | 1 | 0,2 | PREDICTED: vicilin-like antimicrobial peptides 2-2 [Nelumbo nucifera] |
| 27 | 1 | NCBInr | gi\|297835246 | 58 | 26659 | 1 | 1 | 1 | 1 | 0,17 | predicted protein [Arabidopsis lyrata subsp. lyrata] |
| 28 | 1 | NCBInr | gi\|6466096 | 58 | 24429 | 1 | 1 | 1 | 1 | 0,19 | 1-Cys peroxiredoxin [Fagopyrum esculentum] |
| 29 | 1 | NCBInr | gi\|84579412 | 57 | 64534 | 1 | 1 | 1 | 1 | 0,07 | Lactuca sativa 9-cis-epoxycarotenoid dioxygenase 4 |
| 30 | 1 | NCBInr | gi\|227438221 | 57 | 104329 | 1 | 1 | 1 | 1 | 0,04 | disease resistance protein [Brassica rapa subsp. pekinensis] |
| 31 | 1 | NCBInr | gi\|295792020 | 56 | 27630 | 1 | 1 | 1 | 1 | 0,16 | NADH-plastoquinone oxidoreductase subunit 2, partial (chloroplast) [Lygodium japonicum] |
| 32 | 1 | NCBInr | gi\|604312292 | 56 | 49277 | 1 | 1 | 1 | 1 | 0,09 | hypothetical protein MIMGU_mgv1a024803mg, partial [Erythranthe guttata] |
| 33 | 1 | NCBInr | gi\|224061601 | 55 | 54122 | 1 | 1 | 1 | 1 | 0,08 | ethylene-responsive nuclear family protein [Populus trichocarpa] |
| 34 | 1 | NCBInr | gi\|658019417 | 54 | 23924 | 1 | 1 | 1 | 1 | 0,19 | PREDICTED: 21.7 kDa class VI heat shock protein-like [Malus domestica] |
| 35 | 1 | NCBInr | gi\|545370097 | 54 | 93458 | 2 | 2 | 1 | 1 | 0,05 | hypothetical protein COCSUDRAFT_65136 [Coccomyxa subellipsoidea C-169] |
| 36 | 1 | NCBInr | gi\|168044085 | 54 | 106084 | 1 | 1 | 1 | 1 | 0,04 | predicted protein [Physcomitrella patens] |
| 37 | 1 | NCBInr | gi\|596224101 | 54 | 35011 | 2 | 2 | 1 | 1 | 0,13 | hypothetical protein PRUPE_ppa015818mg [Prunus persica] |
| 38 | 1 | NCBInr | gi\|30526289 | 53 | 29454 | 1 | 1 | 1 | 1 | 0,15 | 29 kDa chitinase-like thermal hysteresis protein [Solanum dulcamara] |
| 39 | 1 | NCBInr | gi\|565360693 | 53 | 34277 | 1 | 1 | 1 | 1 | 0,13 | PREDICTED: endonuclease 4-like [Solanum tuberosum] |
| 40 | 1 | NCBInr | gi\|643708794 | 52 | 112251 | 1 | 1 | 1 | 1 | 0,04 | hypothetical protein JCGZ_23543 [Jatropha curcas] |
| 41 | 1 | NCBInr | gi\|565431756 | 52 | 82572 | 1 | 1 | 1 | 1 | 0,05 | hypothetical protein CARUB_v10025969mg [Capsella rubella] |
| 42 | 1 | NCBInr | gi\|661894397 | 51 | 17694 | 1 | 1 | 1 | 1 | 0,27 | unnamed protein product [Coffea canephora] |
| 43 | 1 | NCBInr | gi\|604348300 | 51 | 53260 | 1 | 1 | 1 | 1 | 0,08 | hypothetical protein MIMGU_mgv1a005708mg [Erythranthe guttata] |
| 44 | 1 | NCBInr | gi\|55296895 | 50 | 21876 | 1 | 1 | 1 | 1 | 0,21 | hypothetical protein [Oryza sativa Japonica Group] |
| 45 | 1 | NCBInr | gi\|15226197 | 50 | 79284 | 2 | 2 | 1 | 1 | 0,05 | leucine-rich repeat transmembrane protein kinase-like protein [Arabidopsis thaliana] |
| 46 | 1 | NCBInr | gi\|147841400 | 49 | 66664 | 1 | 1 | 1 | 1 | 0,07 | hypothetical protein VITISV_019908 [Vitis vinifera] |
| 47 | 1 | NCBInr | gi\|470124256 | 49 | 147681 | 1 | 1 | 1 | 1 | 0,03 | PREDICTED: condensin-2 complex subunit D3-like [Fragaria vesca subsp. vesca] |
| 48 | 1 | NCBInr | gi\|698511377 | 48 | 19529 | 1 | 1 | 1 | 1 | 0,24 | PREDICTED: ATP-dependent RNA helicase SUPV3L1, mitochondrial-like [Nicotiana sylvestris] |
| 49 | 1 | NCBInr | gi\|168018115 | 48 | 51118 | 1 | 1 | 1 | 1 | 0,09 | predicted protein [Physcomitrella patens] |
| 50 | 1 | NCBInr | gi\|302788983 | 48 | 34013 | 1 | 1 | 1 | 1 | 0,13 | hypothetical protein SELMODRAFT_443146 [Selaginella moellendorffii] |
| 51 | 1 | NCBInr | gi\|357110912 | 48 | 86292 | 1 | 1 | 1 | 1 | 0,05 | PREDICTED: cell division control protein 48 homolog C [Brachypodium distachyon] |
| 52 | 1 | NCBInr | gi\|15239085 | 48 | 84825 | 1 | 1 | 1 | 1 | 0,05 | pentatricopeptide repeat-containing protein [Arabidopsis thaliana] |
| 53 | 1 | NCBInr | gi\|14423789 | 47 | 7697 | 1 | 1 | 1 | 1 | 0,7 | RecName: Full=Non-specific lipid-transfer protein 2; Short=LTP 2 [Prunus armeniaca] |
| 54 | 1 | NCBInr | gi\|294461233 | 47 | 28036 | 1 | 1 | 1 | 1 | 0,16 | unknown [Picea sitchensis] |
| 55 | 1 | NCBInr | gi\|723456942 | 47 | 22690 | 1 | 1 | 1 | 1 | 0,2 | hypothetical chloroplast RF4 (chloroplast) [Neocystis brevis] |
| 56 | 1 | NCBInr | gi\|147843231 | 47 | 89118 | 1 | 1 | 1 | 1 | 0,05 | hypothetical protein VITISV_019389 [Vitis vinifera] |
| 57 | 1 | NCBInr | gi\|147766466 | 47 | 30370 | 1 | 1 | 1 | 1 | 0,15 | hypothetical protein VITISV_034331 [Vitis vinifera] |
| 58 | 1 | NCBInr | gi\|259161 | 47 | 3007 | 1 | 1 | 1 | 1 | 2,5 | AP24=osmotin homolog {N-terminal} [tomato, Peptide Partial, 27 aa] |
| 59 | 1 | NCBInr | gi\|700253140 | 47 | 157100 | 1 | 1 | 1 | 1 | 0,03 | MSH1, partial [Fragaria vesca] |
| 60 | 1 | NCBInr | gi\|743790641 | 47 | 77619 | 1 | 1 | 1 | 1 | 0,06 | PREDICTED: two-component response regulator-like APRR5 isoform X1 [Populus euphratica] |
| 61 | 1 | NCBInr | gi\|743792320 | 46 | 128334 | 1 | 1 | 1 | 1 | 0,03 | PREDICTED: protein LONGIFOLIA 1-like [Elaeis guineensis] |
| 62 | 1 | NCBInr | gi\|514783407 | 46 | 62278 | 1 | 1 | 1 | 1 | 0,07 | PREDICTED: type I inositol 1,4,5-trisphosphate 5-phosphatase CVP2-like isoform X3 [Setaria italica] |
| 63 | 1 | NCBInr | gi\|695003146 | 46 | 74996 | 1 | 1 | 1 | 1 | 0,06 | PREDICTED: NAC domain-containing protein 78-like isoform X1 [Musa acuminata subsp. malaccensis] |
| 64 | 1 | NCBInr | gi\|226507184 | 46 | 18901 | 1 | 1 | 1 | 1 | 0,25 | Grx_S14 - glutaredoxin subgroup II [Zea mays] |
| 65 | 1 | NCBInr | gi\|657395935 | 44 | 68672 | 1 | 1 | 1 | 1 | 0,06 | cytoplasmic-like arginine-tRNA ligase [Medicago truncatula] |
| 66 | 1 | NCBInr | gi\|15224094 | 44 | 108807 | 1 | 1 | 1 | 1 | 0,04 | putative leucine-rich repeat receptor-like serine/threonine-protein kinase [Arabidopsis thaliana] |
| 67 | 1 | NCBInr | gi\|697120734 | 44 | 17868 | 1 | 1 | 1 | 1 | 0,26 | PREDICTED: uncharacterized protein LOC104107282 [Nicotiana tomentosiformis] |
| 68 | 1 | NCBInr | gi\|672189919 | 44 | 65337 | 1 | 1 | 1 | 1 | 0,07 | PREDICTED: globulin-1 S allele [Phoenix dactylifera] |
| 69 | 1 | NCBInr | gi\|470115872 | 44 | 56899 | 1 | 1 | 1 | 1 | 0,08 | PREDICTED: legumin B-like [Fragaria vesca subsp. vesca] |
| 70 | 1 | NCBInr | gi\|413924608 | 43 | 85140 | 1 | 1 | 1 | 1 | 0,05 | hypothetical protein ZEAMMB73_246912 [Zea mays] |
| 71 | 1 | NCBInr | gi\|12323328 | 43 | 112045 | 1 | 1 | 1 | 1 | 0,04 | unknown protein; 69131-60853 [Arabidopsis thaliana] |
| 72 | 1 | NCBInr | gi\|41584319 | 43 | 24573 | 1 | 1 | 1 | 1 | 0,19 | endo-beta-1,3-glucanase [Glycine tabacina] |
| 73 | 1 | NCBInr | gi\|672127489 | 43 | 157518 | 1 | 1 | 1 | 1 | 0,03 | PREDICTED: formin-like protein 5 [Phoenix dactylifera] |
| 6 | 1 | NCBInr | gi\|729405394 | 84 | 53184 | 1 | 1 | 1 | 1 | 0,08 | PREDICTED: cruciferin PGCRURSE5 [Tarenaya hassleriana] |
| 7 | 1 | NCBInr | gi\|18532 | 79 | 41796 | 1 | 1 | 1 | 1 | 0,11 | actin [Glycine max] |
| 8 | 1 | NCBInr | gi\|565397210 | 73 | 16932 | 1 | 1 | 1 | 1 | 0,28 | PREDICTED: late embryogenesis abundant protein 1-like [Solanum tuberosum] |
| 9 | 1 | NCBInr | gi\|604343782 | 70 | 13662 | 1 | 1 | 1 | 1 | 0,35 | hypothetical protein MIMGU_mgv1a016253mg [Erythranthe guttata] |
| 10 | 1 | NCBInr | gi\|15239136 | 68 | 13272 | 1 | 1 | 1 | 1 | 0,36 | thioredoxin H3 [Arabidopsis thaliana] |
| 11 | 1 | NCBInr | gi\|527205827 | 67 | 76721 | 1 | 1 | 1 | 1 | 0,06 | hypothetical protein M569_03185 [Genlisea aurea] |
| 12 | 1 | NCBInr | gi\|145349412 | 66 | 12318 | 1 | 1 | 1 | 1 | 0,4 | predicted protein [Ostreococcus lucimarinus CCE9901] |
| 13 | 1 | NCBInr | gi\|449462491 | 66 | 147074 | 1 | 1 | 1 | 1 | 0,03 | PREDICTED: pre-mRNA-splicing factor ATP-dependent RNA helicase PRP16-like [Cucumis sativus] |
| 14 | 1 | NCBInr | gi\|302771612 | 66 | 89174 | 1 | 1 | 1 | 1 | 0,05 | hypothetical protein SELMODRAFT_410159 [Selaginella moellendorffii] |
| 15 | 1 | NCBInr | gi\|596224101 | 63 | 35011 | 2 | 2 | 1 | 1 | 0,13 | hypothetical protein PRUPE_ppa015818mg [Prunus persica] |
| 16 | 1 | NCBInr | gi\|693501245 | 62 | 58247 | 1 | 1 | 1 | 1 | 0,08 | unnamed product [Ostreococcus tauri] |
| 17 | 1 | NCBInr | gi\|84579412 | 61 | 64534 | 1 | 1 | 1 | 1 | 0,07 | Lactuca sativa 9-cis-epoxycarotenoid dioxygenase 4 |
| 18 | 1 | NCBInr | gi\|593784427 | 61 | 42506 | 1 | 1 | 1 | 1 | 0,1 | hypothetical protein PHAVU_003G186200g [Phaseolus vulgaris] |
| 19 | 1 | NCBInr | gi\|108710478 | 60 | 88602 | 1 | 1 | 1 | 1 | 0,05 | LOC495012 protein, putative, expressed [Oryza sativa Japonica Group] |
| 20 | 1 | NCBInr | gi\|604348300 | 60 | 53260 | 1 | 1 | 1 | 1 | 0,08 | hypothetical protein MIMGU_mgv1a005708mg [Erythranthe guttata] |
| 21 | 1 | NCBInr | gi\|604312292 | 59 | 49277 | 1 | 1 | 1 | 1 | 0,09 | hypothetical protein MIMGU_mgv1a024803mg, partial [Erythranthe guttata] |
| 22 | 1 | NCBInr | gi\|719968548 | 59 | 22760 | 1 | 1 | 1 | 1 | 0,2 | PREDICTED: vicilin-like antimicrobial peptides 2-2 [Nelumbo nucifera] |
| 23 | 1 | NCBInr | gi\|502161417 | 58 | 48771 | 1 | 1 | 1 | 1 | 0,09 | PREDICTED: cell number regulator 13-like isoform X1 [Cicer arietinum] |
| 24 | 1 | NCBInr | gi\|590665414 | 58 | 72681 | 1 | 1 | 1 | 1 | 0,06 | Uncharacterized protein TCM_012637 [Theobroma cacao] |
| 25 | 1 | NCBInr | gi\|227438221 | 57 | 104329 | 1 | 1 | 1 | 1 | 0,04 | disease resistance protein [Brassica rapa subsp. pekinensis] |
| 26 | 1 | NCBInr | gi\|295792020 | 56 | 27630 | 1 | 1 | 1 | 1 | 0,16 | NADH-plastoquinone oxidoreductase subunit 2, partial (chloroplast) [Lygodium japonicum] |
| 27 | 1 | NCBInr | gi\|297797421 | 55 | 36693 | 1 | 1 | 1 | 1 | 0,12 | hypothetical protein ARALYDRAFT_919715 [Arabidopsis lyrata subsp. lyrata] |
| 28 | 1 | NCBInr | gi\|147790290 | 55 | 20576 | 1 | 1 | 1 | 1 | 0,23 | hypothetical protein VITISV_003045 [Vitis vinifera] |
| 29 | 1 | NCBInr | gi\|657985907 | 55 | 133673 | 1 | 1 | 1 | 1 | 0,03 | PREDICTED: putative disease resistance protein At3g14460 [Malus domestica] |
| 30 | 1 | NCBInr | gi\|545370097 | 54 | 93458 | 2 | 2 | 1 | 1 | 0,05 | hypothetical protein COCSUDRAFT_65136 [Coccomyxa subellipsoidea C-169] |
| 31 | 1 | NCBInr | gi\|566223266 | 54 | 56368 | 1 | 1 | 1 | 1 | 0,08 | hypothetical protein POPTR_0019s01840g [Populus trichocarpa] |
| 32 | 1 | NCBInr | gi\|3080374 | 53 | 93480 | 1 | 1 | 1 | 1 | 0,05 | putative protein [Arabidopsis thaliana] |
| 33 | 1 | NCBInr | gi\|674244918 | 52 | 571656 | 1 | 1 | 1 | 1 | 0,01 | hypothetical protein AALP_AA3G015100 [Arabis alpina] |
| 34 | 1 | NCBInr | gi\|460392935 | 52 | 30818 | 1 | 1 | 1 | 1 | 0,15 | PREDICTED: AT-rich interactive domain-containing protein 2-like [Solanum lycopersicum] |
| 35 | 1 | NCBInr | gi\|566213650 | 52 | 34722 | 1 | 1 | 1 | 1 | 0,13 | hypothetical protein POPTR_0018s01440g [Populus trichocarpa] |
| 36 | 1 | NCBInr | gi\|145344948 | 51 | 17766 | 1 | 1 | 1 | 1 | 0,26 | predicted protein [Ostreococcus lucimarinus CCE9901] |
| 37 | 1 | NCBInr | gi\|470124256 | 51 | 147681 | 1 | 1 | 1 | 1 | 0,03 | PREDICTED: condensin-2 complex subunit D3-like [Fragaria vesca subsp. vesca] |
| 38 | 1 | NCBInr | gi\|729312655 | 51 | 68956 | 1 | 1 | 1 | 1 | 0,06 | PREDICTED: SEC1 family transport protein SLY1-like [Tarenaya hassleriana] |
| 39 | 1 | NCBInr | gi\|565492678 | 50 | 54471 | 1 | 1 | 1 | 1 | 0,08 | hypothetical protein CARUB_v10008910mg [Capsella rubella] |
| 40 | 1 | NCBInr | gi\|12323328 | 50 | 112045 | 1 | 1 | 1 | 1 | 0,04 | unknown protein; 69131-60853 [Arabidopsis thaliana] |
| 41 | 1 | NCBInr | gi\|303277847 | 50 | 21620 | 1 | 1 | 1 | 1 | 0,21 | predicted protein, partial [Micromonas pusilla CCMP1545] |
| 42 | 1 | NCBInr | gi\|731412375 | 50 | 57684 | 1 | 1 | 1 | 1 | 0,08 | PREDICTED: serine carboxypeptidase-like isoform X1 [Vitis vinifera] |
| 43 | 1 | NCBInr | gi\|15239085 | 50 | 84825 | 1 | 1 | 1 | 1 | 0,05 | pentatricopeptide repeat-containing protein [Arabidopsis thaliana] |
| 44 | 1 | NCBInr | gi\|147819109 | 49 | 99818 | 1 | 1 | 1 | 1 | 0,04 | hypothetical protein VITISV_014193 [Vitis vinifera] |
| 45 | 1 | NCBInr | gi\|255080046 | 49 | 52102 | 1 | 1 | 1 | 1 | 0,08 | flagellar radial spoke protein 3 [Micromonas sp. RCC299] |
| 46 | 1 | NCBInr | gi\|595800832 | 49 | 69195 | 1 | 1 | 1 | 1 | 0,06 | hypothetical protein PRUPE_ppa002913mg [Prunus persica] |
| 47 | 1 | NCBInr | gi\|702400075 | 49 | 16885 | 1 | 1 | 1 | 1 | 0,28 | PREDICTED: glycine-rich RNA-binding, abscisic acid-inducible protein-like [Eucalyptus grandis] |
| 48 | 1 | NCBInr | gi\|145349392 | 49 | 73898 | 1 | 1 | 1 | 1 | 0,06 | predicted protein [Ostreococcus lucimarinus CCE9901] |
| 49 | 1 | NCBInr | gi\|695065538 | 48 | 116531 | 1 | 1 | 1 | 1 | 0,04 | PREDICTED: calcium-transporting ATPase 1, endoplasmic reticulum-type-like [Musa acuminata subsp. malaccensis] |
| 50 | 1 | NCBInr | gi\|147843231 | 48 | 89118 | 1 | 1 | 1 | 1 | 0,05 | hypothetical protein VITISV_019389 [Vitis vinifera] |
| 51 | 1 | NCBInr | gi\|629113167 | 48 | 126348 | 1 | 1 | 1 | 1 | 0,03 | hypothetical protein EUGRSUZ_D02334 [Eucalyptus grandis] |
| 52 | 1 | NCBInr | gi\|604335134 | 48 | 31547 | 1 | 1 | 1 | 1 | 0,14 | hypothetical protein MIMGU_mgv1a010985mg [Erythranthe guttata] |
| 53 | 1 | NCBInr | gi\|697120734 | 48 | 17868 | 1 | 1 | 1 | 1 | 0,26 | PREDICTED: uncharacterized protein LOC104107282 [Nicotiana tomentosiformis] |
| 54 | 1 | NCBInr | gi\|674875705 | 47 | 78797 | 1 | 1 | 1 | 1 | 0,06 | BnaA09g03520D [Brassica napus] |
| 55 | 1 | NCBInr | gi\|294461233 | 47 | 28036 | 1 | 1 | 1 | 1 | 0,16 | unknown [Picea sitchensis] |
| 56 | 1 | NCBInr | gi\|723456942 | 47 | 22690 | 1 | 1 | 1 | 1 | 0,2 | hypothetical chloroplast RF4 (chloroplast) [Neocystis brevis] |
| 57 | 1 | NCBInr | gi\|567150070 | 47 | 75927 | 1 | 1 | 1 | 1 | 0,06 | hypothetical protein EUTSA_v10006991mg [Eutrema salsugineum] |
| 58 | 1 | NCBInr | gi\|9187883 | 47 | 80573 | 1 | 1 | 1 | 1 | 0,05 | mitochondrial half-ABC transporter [Arabidopsis thaliana] |
| 59 | 1 | NCBInr | gi\|700253140 | 47 | 157100 | 1 | 1 | 1 | 1 | 0,03 | MSH1, partial [Fragaria vesca] |
| 60 | 1 | NCBInr | gi\|145345262 | 47 | 57518 | 1 | 1 | 1 | 1 | 0,08 | predicted protein [Ostreococcus lucimarinus CCE9901] |
| 61 | 1 | NCBInr | gi\|743792320 | 47 | 128334 | 1 | 1 | 1 | 1 | 0,03 | PREDICTED: protein LONGIFOLIA 1-like [Elaeis guineensis] |
| 62 | 1 | NCBInr | gi\|449464458 | 47 | 12950 | 1 | 1 | 1 | 1 | 0,38 | PREDICTED: thioredoxin H-type-like [Cucumis sativus] |
| 63 | 1 | NCBInr | gi\|695048089 | 47 | 121519 | 1 | 1 | 1 | 1 | 0,04 | PREDICTED: uncharacterized protein LOC103993562 [Musa acuminata subsp. malaccensis] |
| 64 | 1 | NCBInr | gi\|514783407 | 46 | 62278 | 1 | 1 | 1 | 1 | 0,07 | PREDICTED: type I inositol 1,4,5-trisphosphate 5-phosphatase CVP2-like isoform X3 [Setaria italica] |
| 65 | 1 | NCBInr | gi\|15226197 | 45 | 79284 | 1 | 1 | 1 | 1 | 0,06 | leucine-rich repeat transmembrane protein kinase-like protein [Arabidopsis thaliana] |
| 66 | 1 | NCBInr | gi\|147841400 | 45 | 66664 | 1 | 1 | 1 | 1 | 0,07 | hypothetical protein VITISV_019908 [Vitis vinifera] |
| 67 | 1 | NCBInr | gi\|731423405 | 45 | 123378 | 1 | 1 | 1 | 1 | 0,04 | PREDICTED: LOW QUALITY PROTEIN: structural maintenance of chromosomes protein 2-2 [Vitis vinifera] |
| 68 | 1 | NCBInr | gi\|698511377 | 44 | 19529 | 1 | 1 | 1 | 1 | 0,24 | PREDICTED: ATP-dependent RNA helicase SUPV3L1, mitochondrial-like [Nicotiana sylvestris] |
| 69 | 1 | NCBInr | gi\|565450543 | 44 | 42944 | 1 | 1 | 1 | 1 | 0,1 | hypothetical protein CARUB_v10007243mg, partial [Capsella rubella] |
| 70 | 1 | NCBInr | gi\|612396758 | 44 | 32491 | 1 | 1 | 1 | 1 | 0,14 | unknown [Bathycoccus prasinos] |
| 71 | 1 | NCBInr | gi\|674880779 | 44 | 14876 | 1 | 1 | 1 | 1 | 0,32 | BnaC02g47670D [Brassica napus] |
| 72 | 1 | NCBInr | gi\|167997063 | 44 | 26626 | 1 | 1 | 1 | 1 | 0,17 | predicted protein [Physcomitrella patens] |
| 73 | 1 | NCBInr | gi\|672127489 | 43 | 157518 | 1 | 1 | 1 | 1 | 0,03 | PREDICTED: formin-like protein 5 [Phoenix dactylifera] |
